# Understanding the obstacle of incompatibility at residue 156 within HLA-B*35 subtypes

**DOI:** 10.1007/s00251-015-0896-4

**Published:** 2016-01-12

**Authors:** Trishna Manandhar, Heike Kunze-Schumacher, Trevor Huyton, Alexander A. Celik, Rainer Blasczyk, Christina Bade-Doeding

**Affiliations:** Hannover Medical School, Institute for Transfusion Medicine, Feodor-Lynen-Str. 5, 30625 Hannover, Germany

**Keywords:** HLA polymorphism, Peptide-loading complex, Tapasin, Peptide-binding motif

## Abstract

Defining permissive and non-permissive mismatches for transplantation is a demanding challenge. Single mismatches at amino acid (AA) position 156 of human leucocyte antigen (HLA) class I have been described to alter the peptide motif, repertoire, or mode of peptide loading through differential interaction with the peptide-loading complex. Hence, a single mismatch can tip the balance and trigger an immunological reaction. HLA-B*35 subtypes have been described to evade the loading complex, 156 mismatch distinguishing B*35:01 and B*35:08 changes the binding groove sufficiently to alter the sequence features of the selected peptide repertoire. To understand the functional influences of residue 156 in B*35 variants, we analyzed the peptide binding profiles of HLA-B*35:01^156Leu^, B*35:08^156Arg^ and B*35:62^156Trp^. The glycoprotein tapasin represents a target for immune evasions and functions within the multimeric peptide-loading complex to stabilize empty class I molecules and promote acquisition of high-affinity peptides. All three B*35 subtypes showed a tapasin-independent mode of peptide acquisition. HLA-B*35-restricted peptides of low- and high-binding affinities were recovered in the presence and absence of tapasin and subsequently sequenced utilizing mass spectrometry. The peptides derived from B*35 variants differ substantially in their features dependent on their mode of recruitment; all peptides were preferentially anchored by Pro at p2 and Tyr, Phe, Leu, or Lys at pΩ. However, the Trp at residue 156 altered the p2 motif to an Ala and restricted the pΩ to a Trp. Our results highlight the importance of understanding the impact of key micropolymorphism and how a single AA mismatch orchestrates the neighboring AAs.

**Electronic supplementary material:**

The online version of this article (doi:10.1007/s00251-015-0896-4) contains supplementary material, which is available to authorized users.

## Introduction

Hematopoietic stem cell transplantation (HSCT) is widely used as a curative therapy for various hematological malignancies and immune-related disorders (Blazar et al. 2012). Human leucocyte antigens (HLAs) still remain the main barrier for unrelated HSCT, since HLA molecules are characterized by an extensive genetic diversity (Robinson et al. 2015). HLA molecules are scanning the intracellular proteomic content for peptides of self- or non-self origin, bind selected peptides in the peptide-binding region (PBR), and present them to the immune system. Most of the polymorphisms distinguishing allelic variants are located in the PBR, determining the sequence of bound peptides. Every single peptide-HLA complex displays a target for CD8^+^ immune effector cells. It becomes obvious that a stringent HLA matching between donor and recipient is the most important criteria to be considered in transplantation. It could be demonstrated that best outcomes post-transplantation have been achieved using HLA identical sibling donor:recipient pairs, whereas HLA mismatching might lead to serious transplantation complications including graft-versus-host disease (GvHD), graft rejection, and/or mortality (Hauzenberger et al. 2008; Shaw 2008). Rejection episodes occur when the transplanted donor-derived T cells trigger allorecognition of incompatible recipient antigens following HSCT (Goker et al. 2001; Wood and Goto 2012). The chance of finding an identical donor for HSCT is still only 30 % in the Caucasian population (Pidala et al. 2013); hence, in most cases, HLA-mismatched transplants cannot be avoided. Thereby, it is imperative to systematically analyze the differential magnitude of mismatches between HLA subtypes to enable an intelligent mismatching.

Every single HLA-bound peptide alters the accessible surface of the peptide-HLA complex (Burrows et al. 2007) that is presented to immune effector cells; for that reason, the investigation of the individual peptide repertoire and allele-specific peptide-binding profile is the first step to understand and predict histocompatibility. Here, the limiting factors for the selection of a given peptide from the intracellular proteomic pool are (i) the interaction between the HLA and proteins of the peptide-loading complex (PLC) and (ii) the amino acid (AA) composition within the PBR, determining the sequence of a bound peptide. Intracellular peptide loading onto HLA class I molecules in the endoplasmic reticulum (ER) requires the PLC, including the chaperone calreticulin (CRT), thiol-dependent ERp57, transporter associated with antigen processing (TAP), and the HLA class I-dedicated tapasin (TPN) (Ackerman and Cresswell 2004; Wearsch and Cresswell 2008).

TPN is an endoplasmic reticulum (ER) resident transmembrane glycoprotein that exists as a disulfide-linked heterodimer with ERp57 (Dick et al. 2002) and acts in coordination with other components of the PLC to mediate optimal peptide loading, peptide editing, and preferential selection of stable HLA class I complexes (Elliott and Williams 2005; Wearsch and Cresswell 2007). Thus, peptide selection and loading with the assistance of TPN results in the presentation of peptide-HLA complexes with long half-life; most HLA allotypes are highly dependent on TPN for peptide presentation. However, few class I allotypes are able to load peptides independently of TPN; TPN independency is conferred by distinct AA compositions within the HLA heavy chain (hc). A single mismatch at a given position can induce TPN independency. Distinct HLA class I alleles show a unique profile of TPN dependence for peptide loading and cell surface expression, depending on the nature of AAs located on certain polymorphic sites within or outside the PBR (Badrinath et al. 2012; Park et al. 2004). Studies revealed that mismatches located at positions 114 (Park et al. 2003), 116 (Williams et al. 2002), or 156 (Badrinath et al. 2012) in the PBR significantly impact the TPN dependency of certain HLA class I molecules.

Micropolymorphism at residue 156 for HLA-B*44 subtypes has been described to strongly impact on immune responses such as an alteration of the mode of peptide loading (Badrinath et al. 2012), an alteration of the allele-specific peptide repertoire (Fleischhauer et al. 1994), and/or the triggering of T cell alloresponses (Badrinath et al. 2014; Keever et al. 1994). All of these immunological events have the potential to cause graft rejection following HSCT (Fleischhauer et al. 1990).

HLA-B*35 belongs to an allelic group with more than 180 alleles. HLA-B*35 molecules are expressed by approximately 20 % of Caucasians with the most frequent one being HLA-B*35:01^156Leu^ (9 % of Caucasians) (Marsh et al. 2000; Ragupathi et al. 1995). Within the HLA-B*35 group, three naturally occurring allotypes exhibit a single AA exchange at position 156 (HLA-B*35:01^156Leu^, B*35:08^156Arg^, and B*35:62^156Trp^). The allotypes HLA-B*35:01 and B*35:08 have been described to bind peptides of viral (human cytomegalovirus (HCMV) or Epstein-Barr virus (EBV)) origin; the existence of T cells recognizing these pHLA-B*35 could be verified indicating a role of this HLA subtypes in viral immunity. Natural presentation of the described viral peptides by HLA-B*35 alleles has not been observed, yet. However, probable epitopes were mapped using various cell-based assays to demonstrate the function of T cells against the viral pHLA-B*35 complexes (McAulay et al. 2009; Rickinson and Moss 1997). Notably, even though B*35:01 and B*35:08 exclusively differ at residue 156, both alleles bind different peptides. The peptides that can trigger T cell responses when bound to HLA-B*35:01 include HCMV pp65 (IPSINVHHY) (Gavin et al. 1993), EBV EBNA1 (HPVGEADYFEY) (Liu et al. 2014), or EBV BZLF1 (LPEPLPQGQLTAY) (Tynan et al. 2005a). HLA-B*35:08 in complex with HCMV pp65 (CPSQEPMSTYVY) (Wynn et al. 2008), HCMV pp65 (FPTKDVAL) (Wynn et al. 2008), or EBV BZLF1 (LPEPLPQGQLTAY) (Tynan et al. 2005a) elicit strong T cell responses similarly. The ability of these HLA-B*35 alleles to trigger T cell responses when bound to a viral peptide could be suggestive of their possible roles in overcoming the dependence on the classical HLA class I presentation pathway and thus viral immune evasion strategies. In order to understand the structural implication of the 156 mismatch for B*35:01 and B*35:08, crystal structures of HLA-B*35:01^156Leu^ and B*35:08^156Arg^ bound to the same ligand have been solved (Burrows et al. 2007; Green et al. 2004; Tynan et al. 2005b). The distinct structural disparity of these allelic mismatched variants explained their differential peptide selection and consequently their immunogenic incompatibility.

This study was carried out to investigate the functional impact of micropolymorphism at position 156 for HLA*B35 subtypes. We utilized soluble HLA technology (Kunze-Schumacher et al. 2014) in order to analyze the peptide-binding profiles of the three alleles HLA-B*35:01^156Leu^, B*35:08^156Arg^, and B*35:62^156Trp^. The knowledge of allele-specific peptide-binding motifs and repertoires facilitates peptide prediction for vaccination purposes and for defining mismatching scores in transplantation.

## Materials and methods

### Cell lines

The lymphoblastoid cell lines (LCLs) 721.220 (HLA^−^/TPN^−^/TAP^+^), 721.221 (HLA^−^/TPN^+^/TAP^+^), and T2 (HLA^+^/TPN^+^/TAP^−^) were used for transduction of recombinant HLA-B*35/156 constructs. The cell lines were maintained in RPMI 1640 medium (Lonza, Verviers, Belgium) supplemented with 10 % heat-inactivated fetal calf serum (FCS; Lonza, Verviers, Belgium) and 200 mM glutamine (c.c.pro, Oberdorla, Germany). Human embryonic kidney 293 T cells (HEK293T) were cultured in DMEM (Lonza, Verviers, Belgium) supplemented with 10 % heat-inactivated FCS, 20 mM L-glutamine, 1 mg/ml penicillin-streptomycin (c.c.pro, Oberdorla, Germany), and 1 mg/ml G418 (Gibco/Life Technologies GmbH, Darmstadt, Germany). All cell lines were maintained at 37 °C in an atmosphere of 5 % CO_2_.

### Construction of lentiviral vectors

For expression of full length (m) HLA-B*35:01 (exons 1–7) molecules, HLA-B*35:01+ cDNA was amplified by PCR using the primers HLA-B1-TAS (5′-GAGATGCGGGTCACGGCG-3′) and HLA-B-TAAS-E7 (5′-TCAAGCTGTGAGAGACACATCAG-3′). The PCR product was cloned into the lentiviral vector pRRL.PPT.SF.pre.V5-His as previously described (Badrinath et al. 2014). The vectors pRRL.mHLA-B*3508 and pRRL.mHLA-B*3562 were generated by site-directed mutagenesis (SDM) using the primers Sdm_B3508_156_F (5′-GTGGCGGAGCAGCGGAGAGCCTACC-3′) and Sdm_B3508_156_R (5′-GGTAGGCTCTCCGCTGCTCCGCCAC-3′) or Sdm_B3562_156_F (5′-GTGGCGGAGCAGTGGAGAGCCTACCTG-3′) and Sdm_B3562_156_R (5′-CAGGTAGGCTCTCCACTGCTCCGCCAC-3′).

Vectors encoding for soluble (s)HLA-B*35/156 variants were generated from pRRL.mHLA-B*35/156 vectors by introducing a stop codon using the primers Sdm_B35_sE4_F (5′-CCTCACCCTGAGATGAGAGCCATCTTCCCAGTC-3′) and Sdm_B35_sE4_R (5′-GACTGGGAAGATGGCTCTCATCTCAGGGTGAGG-3′).

Verification of HLA-B*35/156 inserts was performed by DNA sequencing using an ABI Prism 3730 DNA Analyzer (Applied Biosystems GmbH, Darmstadt, Germany). Subsequently, endotoxin-free plasmid DNA was purified using the EndoFree^®^ Plasmid Maxi Kit (Qiagen GmbH, Hilden, Germany).

### Recombinant eukaryotic cell lines

LCLs 721.220 and 721.221 or T2 cells were transduced with lentiviral particles encoding for recombinant mHLA-B*35/156 (exons 1–7) or sHLA-B*35/156 (exons 1–4) molecules as described previously (Badrinath et al. 2012). Using Lipofectamine^®^ 2000 (Invitrogen/Life Technologies GmbH, Darmstadt, Germany), HEK293T cells were transfected with the plasmids encoding for mHLA-B*35/156 or sHLA-B*35/156 for production of lentiviral particles. The target cells were transduced with the respective lentivirus encoding for HLA-B*35/156 constructs.

Surface expression of mHLA-B*35/156 molecules was analyzed by flow cytometry using the antibodies anti-bw6-FITC and W6/32-PE; data were acquired on a FACS Canto II (Becton Dickinson GmbH, Heidelberg, Germany).

For quantitative verification of sHLA molecules in the supernatant, a double-antibody sandwich (DAS)-ELISA was applied. The mAb W6/32 was used as a capture antibody and a rabbit anti-human β2m pab was utilized as the detection antibody. The clones with the highest expression of sHLA-B*35/156 molecules were used for large-scale production.

### Large-scale production of sHLA molecules and affinity purification

sHLA-B35*/156 producing B-LCL cells were expanded in the two-compartment bioreactor CELLine classic 1000. Supernatants were harvested weekly and monitored for sHLA production. sHLA molecules were affinity purified using N-hydroxysuccinimide (NHS)-activated HiTrap columns coupled to mAb W6/32. Affinity purification was performed on the BioLogicDuoFlow system (Bio-Rad, Hercules, USA). Trimeric complexes (class I hc, β_2_m, and peptide) were eluted using 0.1 M glycine/HCl buffer (pH 2.7).

### Mass spectrometric analysis of peptides

Following affinity purification, trimeric complexes were filtered through an Amicon Ultra-15 Filter Unit (EMD Millipore/Merck KgaA, Darmstadt, Germany) with a molecular weight cutoff (MWCO) of 10 kDa and the peptides detected in the flowthrough were considered to be of low-binding (LB) affinity. The retentate was further acidified by treatment with 0.1 % trifluoroacetic acid (TFA) and filtered through a MWCO 10-kDa filter to elute high-binding (HB) affinity peptides. Subsequently, the peptides were sequenced using an Eksigent Nano-LC Ultra 2D HPLC coupled to LTQ Orbitrap XL mass spectrometer (Thermo Fisher Scientific GmbH, Schwerte, Germany). The mass spectrometric data were then utilized on Mascot server via a Mascot Daemon interface (www.matrixscience.com). Database queries for peptide sequences and peptide source were carried out by Mascot software (Hirosawa et al. 1993) using the SwissProt 2012_11 human and the respective decoy databases. UniProtKB/Swiss-Prot 2012_11 release of 28 November 2012 contains 538,585 sequence entries, comprising 191,240,774 AAs abstracted from 215,068 references (http://www.uniprot.org).

### Biophysical analysis of HLA-B*35/156 interactions with PLC

Immunoprecipitation experiments were performed in order to analyze the differential interaction of the HLA-B*35/156 hc and distinct PLC components as previously described (Badrinath et al. 2012). mHLA-B*35/156 expressing LCL 721.220 or LCL 721.221 cells were lysed for 30 min on ice in Digitonin lysis buffer. The lysates were collected by centrifugation at 13,000 rpm for 15 min at 4 °C and pre-cleared with protein A-sepharose beads CL-4B for an hour. The lysates were then added to protein A-sepharose beads covalently coupled to rabbit anti-TAP1 pab to capture and immobilize the immune complex on the beads. The immunoprecipitates were analyzed by western blotting using antibodies against the HLA-B hc and selected PLC components.

### Computational analysis

In order to understand the structural implication of the micropolymorphism at position 156 on HLA-B*35 molecules, a computational simulation was performed to artificially exchange AAs at position 156. The structure of HLA-B*35:01 (2AXG) (Tynan et al. 2005b) was overlaid with that of B*35:08 (2AXF) (Tynan et al. 2005b), and a model of B*35:62 was generated by mutation of Leu at 156 (B*35:01, 2AXG) to Trp using YASARA/FoldX software.

## Results

### Peptide recruitment and biophysical association with the PLC

**Fig. 1:**
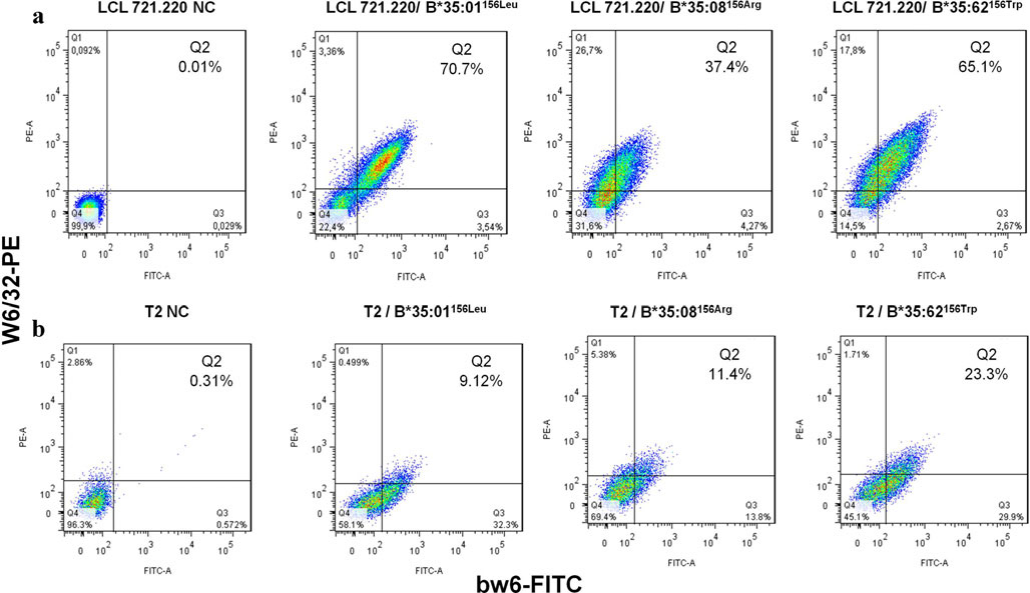
mHLA-B*35/156 expression. Cells were analyzed for surface expression of mHLA complexes using anti-bw6-FITC and anti-HLA-A/B/C-PE (W6/32-PE) monoclonal antibodies. **a** Surface expression of mHLA-B*35/156 variants on LCL 721.220 (HLA^−^/TPN^−^/TAP^+^) cells. **b** Surface expression of mHLA-B*35/156 variants on T2 (HLA^−^/TPN^+^/TAP^−^) cells. A higher amount of molecules could be detected for mHLA-B*35:01 and B*35:62 on LCL 721.220 cells in comparison to the amount of B*35:08 molecules. Results show relatively higher surface expression of HLA-B*35:62 compared to B*35:01 and B*35:08 on T2 cells, thereby indicating that the surface expression of B*35:62 is comparatively more independent of TAP. Annotations: *NC* negative control (untransduced cells)

Flow cytometric analysis showed differential surface expression of mHLA-B*35/156 molecules on the surface of LCL 721.220 or 721.221 cells. The investigated B*35/156 variants were able to load peptides independently of TPN; however, the density of mHLA-B*35/156 molecules on the cell surface is clearly influenced by the type of polymorphism at residue 156. The expression of mHLA-B*35:08 was found to be comparatively low in the absence of TPN compared to HLA-B*35:01 and B*35:62 (Fig. 1a). These data illustrate that the surface expression of HLA-B*35:08 is relatively more TPN dependent than the surface expression of the other allelic variants investigated. Results demonstrated that the surface expression of mHLA-B*35:62 was relatively more independent of TAP compared to those of mHLA-B*35:01 and B*35:08 (Fig. 1b).

**Fig. 2:**
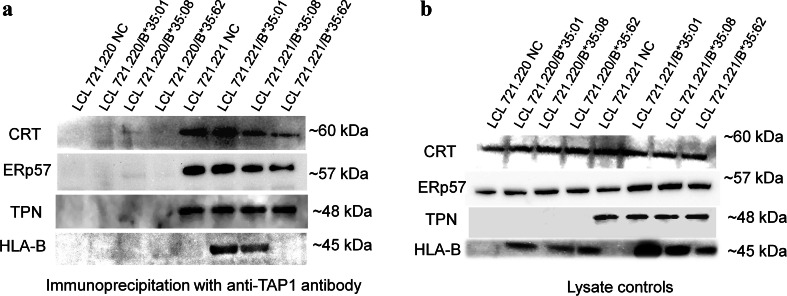
Biophysical interaction of mHLA-B*35/156 with PLC components. Cell lysates were used for immunoprecipitation with an anti-TAP1 antibody. Protein-protein interactions were detected with antibodies against HLA-B hc, CRT, ERp57, and TPN. **a** Detection of CRT, ERp57, TPN, and HLA-B hc in TAP1-precipitates. **b** Lysate controls showing levels of CRT, ERp57, TPN, and HLA-B hc in untransduced and transduced LCL 721cells. The presence of essential PLC components in the cells could be verified in the lysate controls. In cells lacking TPN, an interaction between the HLA-B*35/156 hc and TAP was not detected. In TPN-competent cells, hcs of HLA-B*35:01 or B*35:08 were strongly associated with TAP while no such association could be demonstrated with B*35:62 hc

The association of mHLA-B*35/156 molecules and TAP could not be detected in TPN-deficient cells, indicating the significance of TPN in bridging TAP and the HLA molecule. However, a strong association of mHLA-B*35:01 and B*35:08 with TAP was demonstrated in TPN-competent cells. Surprisingly, no such interaction could be detected for mHLA-B*35:62 (Fig. 2), indicating its complete PLC-independent mode of peptide loading.

### Peptide profiling

**Fig. 3:**
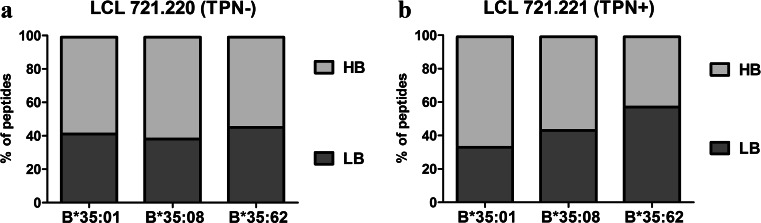
Differential stability of sHLA-B*35/156 molecules. The *x* axis represents the HLA-B*35 subtypes, while the *y* axis represents percentage prevalence of LB and HB peptides. *Dark grey* represents the LB peptides and *light grey* the HB peptides. **a** Analysis of total LB and HB peptides acquired in LCL 721.220 cells. **b** Analysis of total LB and HB peptides acquired in LCL 721.221 cells. sHLA-B*35:62 molecules derived from LCL 721.221 cells showed a higher appearance (57.63 %) of LB than HB peptides. In contrast, sHLA-B*35:01/35:08 molecules revealed the presentation of comparatively higher percentages of HB peptides than LB peptides

A total of 229, 261, or 492 peptides restricted to sHLA-B*35:01, B*35:08, or B*35:62 are given in Table 1; peptide sequences are given in Supplementary 1. Although an equal concentration of purified sHLA-B*35/156 molecules was utilized for mass spectrometric analysis of the bound peptides, differential numbers of peptides could be recovered due to differential pHLA complex stability among HLA-B*35/156 variants. A comparative analysis of HB and LB peptides facilitates the association of peptide-binding strength and distinct AA exchanges in the HLA hc. The individual profile of peptides acquired in the presence or absence of TPN showed relatively higher numbers of HB peptides for HLA-B*35:01 and B*35:08 (Fig. 3). In contrast, more LB peptides could be recovered from sHLA-B*35:62 molecules in the presence or absence of TPN.

**Table 1:** Profile of peptides

HLA-B*35 allele	HB/LB peptides	Peptides (*N*)
B*35:01 (TPN^−^)	LB	38
	HB	54
B*35:01 (TPN^+^)	LB	46
	HB	91
B*35:08 (TPN^−^)	LB	33
	HB	53
B*35:08 (TPN^+^)	LB	76
	HB	99
B*35:62 (TPN^−^)	LB	117
	HB	139
B*35:62 (TPN^+^)	LB	136
	HB	100

This table shows the overall profile of LB or HB peptides

*LB* low-binding peptides, *HB* high-binding peptides, *TPN* ^+^ acquired in the presence of TPN, *TPN* ^−^ acquired in the absence of TPN, *N* number of peptides

### Shared peptides

**Fig. 4:**
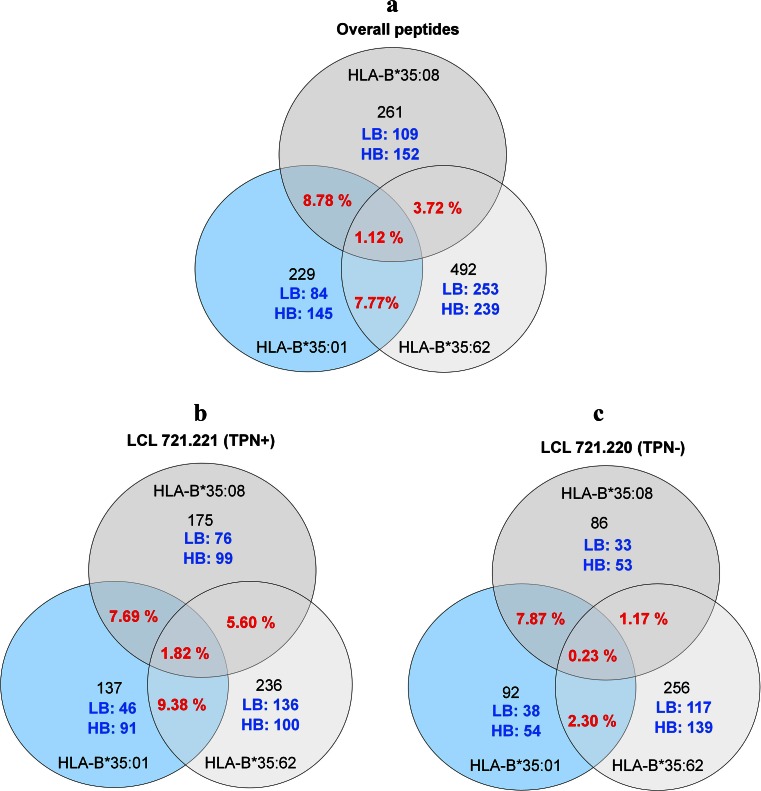
Shared peptides of sHLA-B*35/156 variants. **a** Overall shared peptide repertoire of HLA-B*35:01, B*35:08, or B*35:62 restricted peptides. **b** Shared peptides acquired in the absence of TPN. **c** Shared peptides acquired in the presence of TPN. A small percentage (1.12 %) of the overall peptide repertoire was found to be shared between the three different allotypes. Among the pool of peptides, 0.23 and 1.82 % of eluted peptides were shared in the absence and presence of TPN, respectively

sHLA-B*35/156 variants were expressed in LCL 721.220 or 721.221 cells to ensure that the proteomic content is virtually identical, with the major difference that in 721.220 cells, the peptides are loaded without the assistance of TPN. However, the HLA-B*35/156 allotypes were found to share a very small subset of their overall peptide repertoire, both in the presence and absence of TPN. Shared peptide analysis shows that 11 peptides (1.12 %) were shared among the HLA-B*35/156 variants (Fig. 4). More peptides were shared in the presence of TPN than in its absence. Among these shared peptides, a 12-mer, ALSTGEKGFGYK (peptidyl-prolylcis-trans isomerase A) was acquired in the absence of TPN, while 10 peptides were acquired in the presence of TPN (Fig. 4b, c). The majority of overall shared peptides associated with HLA-B*35:01 or HLA-B*35:08 were HB peptides (81.81 % for HLA-B*35:01 and 63.63 % for HLA-B*35:08), while only 18.18 % of shared peptides associated with HLA-B*35:62 were found to be HB peptides (Supplementary 2).

Only 3.72 % of the peptides were shared between HLA-B*35:08 and HLA-B*35:62, while 7.77 % were shared between HLA-B*35:01 and B*35:62. HLA-B*35:01 and B*35:08 share an overall peptide repertoire of 8.78 % (Fig. 4a). Between the three allelic variants, a highly variable peptide repertoire could be observed; it becomes obvious how the differential peptide-loading pathways lead to a divergent selection of the proteomic content. The differential association of the HLA-B*35 allelic variants with the loading complex reflects on the peptide selection. This is highlighted by the numbers of shared peptides given in Fig. 4.

### Characteristics of peptide anchor positions

**Fig. 5:**
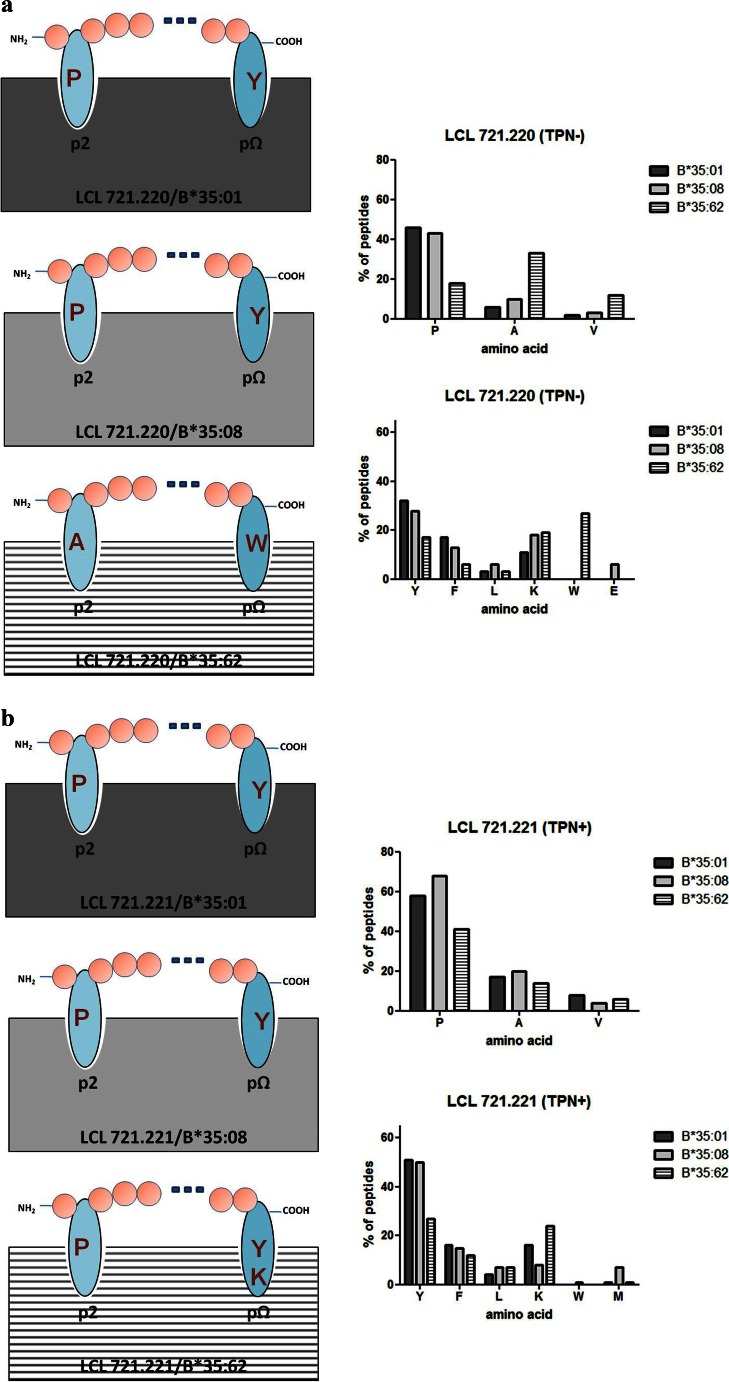
p2 and pΩ anchor positions of sHLA-B*35/156-restricted peptides. *Characters in red* represent the predominant AAs occurring at major anchor position (frequencies >25 %). The *dots* indicate the variable number of AA residues. The *graphs on the right panel* indicate the frequencies of AAs. The *x* axis represents the AA residues at p2 and pΩ. The *y* axis represents the percentage prevalence of individual AAs. *Black*, *grey*, or *crossed bars* represent the alleles HLA-B*35:01, B*35:08, or B*35:62. **a** Peptide anchor motif in the absence of TPN. **b** Peptide anchor motif in the presence of TPN. Pro was the most frequently occurring AA at p2 position. All HLA-B*35/156 variants are predominantly anchored by Tyr, Phe, Leu, or Lys at pΩ. However, HLA-B*35:62 is preferentially anchored by Ala at p2 and Trp at pΩ in the absence of TPN

Analysis of HLA-B*35/156-restricted peptides demonstrated Pro at p2 (Fig. 5). The preference for Pro at p2 was decreased for HLA-B*35:01-restricted LB peptides that were acquired in the presence of TPN. HLA-B*35:08 and B*35:62 were additionally anchored at p2 by Ala and Val. HLA-B*35:62-restricted peptides that were acquired in the absence of TPN were exclusively anchored by Ala at p2 (Fig. 5a).

At pΩ, a preference for Tyr, Phe, Leu, or Lys could be observed (Fig. 5). Unlike HLA-B*35:01 and B*35:08, B*35:62 preferentially demonstrated Trp at pΩ when acquired in the absence of TPN. Approximately 50 % of sHLA-B*35:62-restricted HB peptides acquired in the absence of TPN exhibited a preference for Trp at pΩ (Supplementary 3). Furthermore, 13.13 % of sHLA-B*35:08-restricted HB peptides acquired in the presence of TPN were preferentially anchored by Met at pΩ.

### Length distribution of peptides

**Fig. 6:**
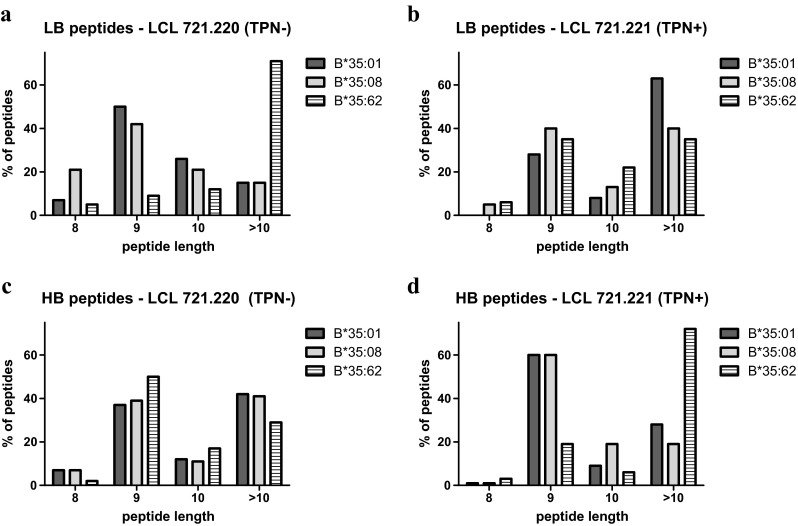
Length distribution of sHLA-B*35/156-restricted peptides. Peptide length (AA) is given on the *x* axis, and the percentage prevalence of peptides is given on the *y* axis. *Black*, *grey*, or *crossed bars* represent the alleles HLA-B*35:01, B*35:08, or B*35:62. **a**, **b** Length distribution of LB peptides. **c**, **d** Length distribution of HB peptides. In the presence of TPN, the majority of HLA-B*35:01- and B*35:08-restricted HB peptides were of canonical length. HLA-B*35:62-restricted LB peptides that were acquired in the absence of TPN were of non-canonical length

The majority of sHLA-B*35/156-restricted peptides were found to be of canonical length (8–10 AAs). However, peptides of non-canonical length (>10 AAs) could also be recovered by sHLA-B*35/156 variants (Fig. 6). HLA-B*35:62, in particular, was found to preferentially present peptides of non-canonical length (48.83 and 50.85 % in the absence and presence of TPN, respectively). Among the peptides presented, 31.52 and 31.40 % non-canonical peptides were presented by HLA-B*35:01 and B*35:08, respectively, in the absence of TPN. In the presence of TPN, HLA-B*35:01 was shown to present 40.15 % non-canonical peptides, while HLA-B*35:08 was shown to present a lesser percentage of longer peptides.

A significant percentage of HB peptides recovered from HLA-B*35:01 or B*35:08, in the presence of TPN, was found to be of canonical lengths (71.43 % for HLA-B*35:01 and 80.81 % for HLA-B*35:08); this is in contrast to HLA-B*35:62-restricted peptides (28 %).

Taken together, in the absence of TPN, both canonical and non-canonical sHLA-B*35/156-restricted HB peptides were observed. The majority of HLA-B*35:01- and B*35:08-restricted LB peptides were of canonical lengths. Contrary, most of the HLA-B*35:62-restricted LB peptides acquired in the absence of TPN were of non-canonical length (71.79 %).

### Molecular modeling

**Fig. 7:**
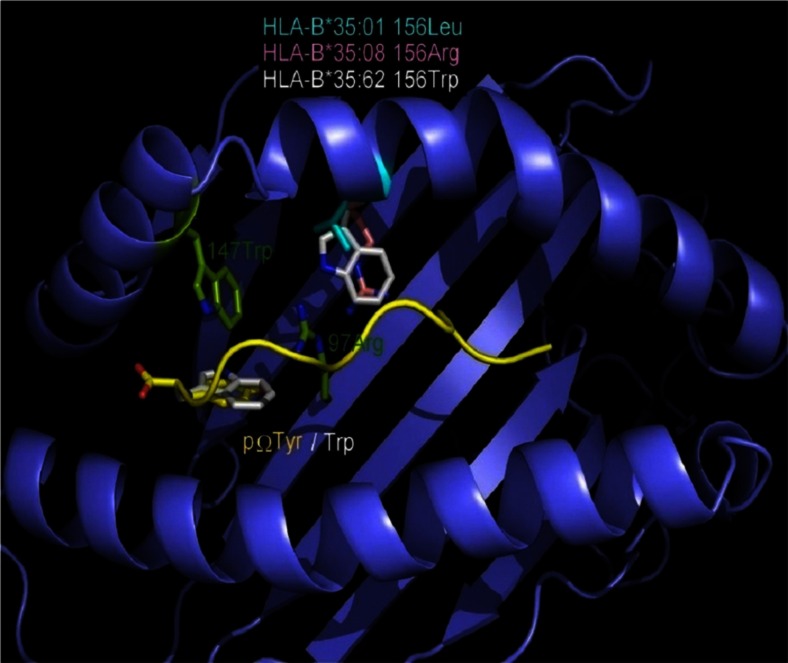
Model of HLA-B*35:62. Molecular modeling of position 156 in HLA-B*35 utilizing YASARA/FoldX software. Structural overlay of HLA-B*35:01 and B*35:08 both bound to a decamer peptide (APQPAPENAY) and the modeled structure of HLA-B*35:62. PDB, 2AXG (Tynan et al. 2005b) and 2AXF (Tynan et al. 2005b)

sHLA-B*35:62-restricted peptides that were acquired in the absence of TPN are predominantly anchored by a C-terminal Trp. Residue 156 that distinguishes the three allelic variants is not part of pocket F and thus has no direct influence on the peptide C-terminus. To understand the unexpected alteration of the C-terminal peptide anchor, YASARA/FoldX software was utilized for generating a model of HLA-B*35:62. Crystal structures of HLA-B*35:01 (2AXG) (Tynan et al. 2005b) and B*35:08 (2AXF) (Tynan et al. 2005b) are available and were overlayed. Since no structure of HLA-B*35:62 is available, a model of HLA-B*35:62 was generated by mutating 156Leu in HLA-B*35:01 to 156Trp. In the HLA-B*35:62 model, the stacking arrangement of 147Trp and 156Trp against 97Arg alters the F pocket indirectly and allows for a C-terminal Trp of the bound peptides (Fig. 7).

## Discussion

The degree of donor:recipient HLA-compatibility strongly influences clinical outcome after unrelated stem cell transplantation. Considerable efforts have been made over the last years to distinguish non-permissive HLA mismatches that dramatically increase the risk of post-transplantation outcomes from permissive HLA mismatches (Bacigalupo 2013; Flomenberg et al. 2004). Understanding the impact of a particular key polymorphism on the mode of peptide loading, the repertoire of selected and presented peptides, and consequently the alteration in pHLA landscapes and half-life is the pre-requisite for the estimation of mismatch allogenicity when no clinical data are available.

The loading of peptides onto HLA class I molecules is a complex procedure and facilitated by the PLC, wherein TAP and TPN are essential components. TPN supports peptide loading, stabilizes the TAP complex, and thus indirectly promotes the accessibility of peptides in the ER (Garbi et al. 2003). Furthermore, TPN is known to function in peptide editing and loading of high-affinity peptides (Barnden et al. 2000; Williams et al. 2002). In the absence of TPN, interaction of class I molecules to TAP is disrupted, resulting in unstable pHLA complexes (Peh et al. 2000). Most of the HLA class I molecules rely strictly on the functions of TPN and TAP for efficient peptide loading. Certain viral proteins and cancer proteins target these PLC components to prevent the presentation of immunogenic peptides and recognition by cytotoxic CD8^+^ T cells. Nevertheless, recent studies document host strategies to circumvent these immune evasions by choosing pathways (Boyle et al. 2013; Fromm et al. 2002; Lautscham et al. 2003) that could operate independent of these PLC components. Certain allelic HLA class I variants that load peptides independently from TPN and/or TAP are therefore still able to present a fraction of the intracellular peptide repertoire to the immune system. However, TPN- or TAP-independent peptide presentation might lead to a differential peptide repertoire that would be selected and subsequently presented, since those peptides are not optimized for stabilizing the respective HLA allele. HLA class I variants that are TPN- and/or TAP-independent differ from the PLC-dependent alleles often exclusively by one AA difference (Badrinath et al. 2012; Neisig et al. 1996; Williams et al. 2002). Understanding the effect of polymorphism on the immune function of a given HLA molecule can only take the form of a measure of histocompatibility. The similarity of allele peptide-binding profiles and repertoires is such a measure.

HLA-B*35:01 and B*35:08 can be distinguished by a micropolymorphism at position 156; both variants are described to present viral peptides (Liu et al. 2014; Tynan et al. 2005a, 2007; Wynn et al. 2008), suggesting a PLC independency for these allelic variants. This study was focused on determining the mode of antigen recruitment utilized by natural occurring HLA-B*35 allotypes that differ at one single mismatch at residue 156, HLA-B*35:01^156Leu^, B*35:08^156Arg^, and B*35:62^156Trp^.

All three HLA-B*35/156 variants studied were expressed on the cell surface of TPN-deficient cells, suggesting a TPN-independent mode of peptide acquisition and presentation for these molecules. However, the spectrum of TPN dependence among these allotypes was found to vary with the nature of AA at position 156. The data revealed the influence of microploymorphism at position 156 on the molecule expression levels and stability of pHLA complexes on the cell surface. A recent study by Rizvi et al. (2014) also demonstrated a TPN-independent mode of peptide loading for HLA-B*35:01. The 156 micropolymorphism distinguishing the HLA-B*35/156 allotypes causes a functional disparity for their TPN interaction. Unlike HLA-B*35:01^156Leu^ and B*35:62^156Trp^, surface expression of B*35:08^156Arg^ was decreased by more than twofold in the absence of TPN, indicating partial dependence of B*35:08 on TPN for peptide loading and presentation. In addition to TPN independence, our results showed that the surface expression of HLA-B*35:62 was relatively independent of TAP compared to that of HLA-B*35:01 and B*35:08.

Immunoprecipitation experiments demonstrated differential association of TAP with the HLA-B*35:01^156Leu^ hc, B*35:08^156Arg^ hc, or the B*35:62^156Trp^ hc. The HLA-B*35/156 hc could not be detected in any of the TAP complexes from cells lacking TPN (LCL 721.220 cells), since the association between TAP and the HLA hc is mediated through TPN. TPN plays an important role in steady state expression of TAP (Lehner et al. 1998) and also helps in the stabilization of the heterodimeric TAP1/TAP2 complex. In contrast, in TPN positive cells (LCL 721.221), a strong association of both HLA-B*35:01 hc and HLA-B*35:08 hc with TAP could be detected. The observations validate the accepted role of TPN in bridging a HLA class I molecule and the TAP complex (Ortmann et al. 1997; Sadasivan et al. 1996). However, unlike HLA-B*35:01 and B*35:08, the association of the B*35:62 hc with TAP could not be confirmed. This result could mean that either HLA-B*35:62 does not incorporate into the PLC and does not utilize the TAP complex for peptide loading or the transit period of HLA-B*35:62 into the PLC is so concise that the interaction could not be detected using this experimental approach.

The results from the immunoprecipitation experiments were in synchrony with the flow cytometric studies where the surface expression of HLA-B*35:62 was found to be comparatively independent of TAP. The observed results could be suggestive that HLA-B*35:62 might be very weakly or not at all associated with TAP. The findings postulate the involvement of an unknown alternate pathway for peptide selection and presentation by HLA-B*35:62. Given that all the three HLA-B*35/156 variants share the same AA sequence except for the single AA polymorphism at position 156, Trp156 in HLA-B*35:62 would be the most likely factor regulating the association of this allele with TAP and modulating a differential mode of peptide loading. Several studies revealed how certain allelic variants that differ at one or more AAs might vary in their TAP association. HLA-B*44:02^116Asp/156Asp^ which differs from HLA-B*44:03^116Asp/156Leu^ by a single AA at position 156 and from HLA-B*44:05^116Tyr/156Asp^ at position 116 bounds strongly to TAP, while HLA-B*44:03^116Asp/156Leu^ (Neisig et al. 1996) and HLA-B*44:05^116Tyr/156Asp^ (Zernich et al. 2004) did not. It was demonstrated that HLA-A*68:07^116His/70Gln^ is associated much stronger with TAP than HLA-A*68:03^116Asp/70His^ (Turnquist et al. 2002). In 1996, Neisig et al. (1996) demonstrated that HLA-B alleles with aromatic AAs at position 116 could be a better TAP binder compared to the others. It was observed that among the HLA-B*15 allotypes, HLA-B*15:10^116Tyr^ showed a stronger association with TAP compared to HLA-B*15:18^116Ser^ (Turnquist et al. 2000). In case of HLA-B*35:62^156Trp^, it is possible that Trp at position 156 alters the overall conformation of the PBR sufficiently to affect its interaction with neighboring AA residues, thereby rendering a TAP-independent mode of peptide loading.

To analyze if the polymorphic difference at position 156 alters the peptide-binding specificities, soluble HLA technology (Bade-Doeding et al. 2004; Kunze-Schumacher et al. 2014) was utilized to characterize the repertoire of peptides presented by HLA-B*35:01^156Leu^, B*35:08^156Arg^, and B*35:62^156Trp^. In the present study, it could be demonstrated how a single mismatch at residue 156 in HLA-B*35/156 allotypes changes the peptide-binding groove sufficiently to alter the features of the selected peptide repertoire.

Position p2 and the C-terminal position pΩ of a peptide are significant for an effective binding in the PBR of most allotypes (Falk et al. 1991; Parker et al. 1992) and thus determine allelic specificity. Hence, the HLA-B*35/156-bound peptides were investigated for the peptide-binding anchor motifs at p2 and pΩ. The results demonstrated similar peptide-binding preferences for HLA-B*35:01 and B*35:08; however, HLA-B*35:62 showed striking differences for the anchor motif at p2 and pΩ, especially in TPN-deficient cells. The mass spectrometric analysis revealed that HLA-B*35:01- and B*35:08-restricted peptides acquired in the presence or absence of TPN are preferentially N-terminal anchored by Pro at p2. The preference for Pro at p2 for HLA-B*35:01 and B*35:08 is consistent with previous studies (Escobar et al. 2008; Falk et al. 1993; Sidney et al. 1995; Steinle et al. 1995). In contrast, surprisingly, the binding motif of HLA-B*35:62 showed an unusual preference for Ala at p2, in the absence of TPN. Comparison of AA frequencies in peptides derived from sHLA-B*35/156 showed a preference of Tyr, Phe, Leu, or Lys at pΩ. Unlike HLA-B*35:01 and B*35:08, B*35:62 preferentially binds peptides with Trp at pΩ in the absence of TPN. These results imply the influence of Trp at position 156 in HLA-B*35:62 on alteration of peptide selectivity by TPN. To validate the possible structural implication of position 156 on HLA-B*35:62 molecule, a structural model of HLA-B*35:62 was generated by mutating Leu at position 156 in the HLA-B*35:01 structure (2AXG) (Tynan et al. 2005b) to Trp. This mutational model of HLA-B*35.62 revealed a stacking arrangement of 147Trp and 156Trp against 97Arg. The residue triad, 147Trp/156Trp/97Arg, was found to be highly selective for a C-terminal Trp of the bound peptide. It was also demonstrated that HLA-B*35/156 variants were able to present peptides of non-canonical lengths (>10 AAs). Generally, it was considered that HLA class I molecules present peptides of canonical length (8–10 AAs). The limit for peptide length restriction depends, in part, by structure and conformation of PBR. It was reported that peptides of non-canonical length could be bound by HLA-B*35:01 and B*35:08 molecules and such peptides can be highly immunogenic (Green et al. 2004; Probst-Kepper et al. 2004; Tynan et al. 2005a).

Furthermore, to understand if the micropolymorphism at position 156 would impact the stability of pHLA complexes, peptide binding affinities, as reflected by profiles of LB and HB peptides, were analyzed and correlated with the results of flow cytometry and immunoprecipitation experiments. Analysis of peptides presented by HLA-B*35:01 and B*35:08 molecules in the presence of TPN (LCL 721.221 cells) displayed the majority of them being HB peptides. It was expected that the absence of TPN would lead to the presentation of a higher quantity of LB peptides. However, no significant differences between the percentages of HB and LB peptides were observed from peptides presented by these alleles in the absence of TPN. Especially, it was expected that HLA-B*35:08, which was found to be relatively more dependent on TPN for surface expression, would present peptides of low affinity in the absence of TPN. Our findings suggests that the probable dependence of HLA-B*35:08 on TPN is on egress of pHLA complex from ER to cell surface and their stabilization on cell surface rather than inside the cell. The results could be comparable to the findings observed in peptides associated with HLA-B*08:01 and HLA-A*02:01, where the peptides acquired in absence of TPN were found to be of unexpected higher affinity than those acquired in its presence (Zarling et al. 2003). Moreover, Raghuraman et al. (2002), using an insect cell-reconstituted system and peptide translocation assay, observed that TPN does not alter the peptide translocation efficiency and presence of TPN unexpectedly slightly reduced the affinity of TAP complexes for peptides, suggesting that TPN is less likely to alter the peptide selectivity by TAP and hence the features of TAP-translocatable peptides. These results indicated the role of TPN in stabilizing a peptide receptive conformation of the PBR but not to function as a peptide editor to discriminate between low- and high-binding peptides. Furthermore, a peptide receptive conformation of the PBR may not always mean the conformation of HLA molecule with HB affinity or tightly bound peptides.

Beside this, HLA-B*35:62 was shown to present a higher percentage of LB peptides in the presence of TPN that could be indirectly attributed to its TAP-independent character. The selectivity of TAP is important because it helps in peptide selection by translocation of peptides of optimal length and sequence to their corresponding HLA class I molecules (Momburg et al. 1994a, b). Our experimental data revealed that HLA-B*35:62 could acquire peptides independent of TAP. It is most likely that TAP is not being utilized for selection of HLA-B*35:62-specific peptides; therefore poorly selected peptides are presented by B*35:62 even in the presence of TPN. All these observations imply that the AA mismatch at position 156 in HLA-B*35 variants has the potential to alter the stability of these pHLA complexes and influence the mode by which TPN or TAP functions.

The peptide-binding characteristics of individual HLA class I proteins are shown to be a major factor determining the immunorecognition of pathogens (Pereyra et al. 2010) and the findings for these HLA-B*35/156 alleles indicate a probable role in the presentation of viral epitopes. Moreover, refolding assays on conformational stabilities have shown that TPN-independent allotypes were found to be more assembly competent and are in a more stable peptide-receptive conformation compared with TPN-dependent allotypes (Geironson et al. 2013; Rizvi et al. 2014). This result highlighted the added advantage of these TPN-independent alleles for pathogen recognition. Paradoxically, dependence of individual HLA class I molecules on TPN can influence the assembly and stability of individual HLA class I molecules and have a subsequent impact on disease progression (Rizvi et al. 2014).

Moreover, the presence of Trp at position 156 in HLA-B*35:62 was shown to confer a TAP-independent mode of peptide loading that could be suggestive of conferring the ability of peptide presentation via non-classical pathways and its potential role in immune response against viral infections. However, such differences on the mode of peptide loading can have interference on the alliance with ER quality control factors, stabilities of antigenic peptide associations with HLA-B molecules, and hence the abilities of HLA-B molecules to mediate immune responses during infections. HLA-B*35:62 is a rare allele occurring sparsely in Hispanic population (http://www.allelefrequencies.net; http://www.ebi.ac.uk) and further studies are needed to better understand the reason for its failure to be selected in the course of host-pathogen co-evolution.

Our study highlights how a single AA mismatch at position 156 orchestrates the mode of peptide loading and repertoire of presented peptides. This study as a whole is a part of our continual effort to provide better explanation and estimation of outcomes of HLA mismatches through biochemical and structural analyses of key polymorphic position in HLA alleles. These results will direct toward intelligent mismatching strategies and guide toward personalized treatment of viral infections.

## Electronic supplementary material

ESM 1 (DOCX 118 kb)

ESM 2 (DOCX 23 kb)

ESM 3 (DOCX 226 kb)
