# Supplementary material for: Understanding the obstacle of incompatibility at residue 156 within HLA-B*35 subtypes
Source: Immunogenetics. 2016 Jan 12;68:247–60. doi: 10.1007/s00251-015-0896-4 (PMC4799800; doi:10.1007/s00251-015-0896-4)
Supplement: Supplementary file 1 — (DOCX 118 kb) [file 251_2015_896_MOESM1_ESM.docx]

**Supplementary materials**

**Supplementary 1**

**List of peptides eluted from cells transduced with constructs encoding for sHLA-B*35/156 molecules**

**Low binding peptides associated with HLA-B*35:01 (origin LCL 721.220 cells)**

Peptide position

1 2 3 4 5 6 7 8 9 10 11 12 13 14 15 16 17 18 19 20 21

Ligands Source

E A F L N N Q Y Spectrin beta chaon

P A G VV N K Y 7-dehyd. Cholesterol reductase

M A L A D S A I Insulin receptor substrate 4

M P E D V K N F Y Tubulin polyglutamylase complex subunit 2

Q P M E V Q E G Y Transcription intermediary factor 1-beta

L P D T R S E A Y Cyclin-T1

M P T T G I N E Y Guanine nucleotide-binding prot. subunit alpha-15

L A L V T L L S F Prot. O-mannosyl-transferase 2

V P V G E K T T Y Melanotransferrin

Y P T Q P G Q G Y RNA-binding prot. FUS

I P V T I I T G Y COBW domain-containing prot. 1

L P P P P P G S F Mitogen-activated prot. kinase kinase kinase 5

L P Q D V I L K F Probable ATP-dependent RNA helicase DDX27

L P D E A S E A F Huntingtin

I A L K A V T N F Bromodomain-containing prot. 7

E A A V A I K A M Eukaryotic translation initiation factor 5A-1-like

M P Q G A P R L Y Phosphatidylinositol 3,4,5-trispho-dep Rac prot.

F P D K P I T Q Y Dolichyl-diphosphooligosaccharide—prot.

Y P V D L G D K F DNA-directed RNA polymerases I, II, and III subunit RPABC3

M P A D T N K A F ER membrane prot. complex subunit 3

L P N D G D E K Y Programmed cell death prot. 2-like

T P A G V V N K Y 7-dehydrocholesterol reductase

Y P A P E R L Q E Y Putative WAS prot. family homolog

H P I I P E Q S T F Suppressor of G2 allele of SKP1 homolog

F P E T T S P H E Y Neuroblastoma-amplified sequence

L P I D P N E P T Y Inhibitor of growth prot. 1

H P L S L T S D Q Y Interferon regulatory factor 3

I P I A G R D I T Y Actin-related prot. 3B

S P A Q E D G K V Y Hematopoietic cell signal transducer

Y K C V S C T K T F Zinc finger prot. 668

L P S P V T A Q K Y Elongation factor 2

F P M T H G N T G F Poly(rC)-binding prot.

T P I Q D N V D Q T Y Germinal center-associated signaling and motility prot.

M L N I V Q D S A L L Ral GTPase-activating prot. subunit beta

M P V A A R E A S I Y V-type proton ATPase catalytic subunit A

S P V N S S K Q P S Y ATP synthase lipid-binding prot., mitochondrial

I P Y H S E V P V S L Spectrin beta chain, non-erythrocytic 1

D P A P L G A G N L G P Prot. DPCD

**High binding peptides associated with HLA-B*35:01 (origin LCL 721.220 cells)**

Peptide position

1 2 3 4 5 6 7 8 9 10 11 12 13 14 15 16 17 18 19 20 21

Ligands Source

T G E G F Y K Y Hydroxyacyl-coenzyme A dehydrogenase, mitochondrial

I Q K F P V G R Transmembrane protease serine 9

P A Q Y P P P P RNA binding prot. fox-1 homolog 3

N E E N I F I I Protocadherin-18

H P T I I S E S F T-complex prot. 1 subunit delta

H P T S V I S G Y T-complex prot. 1 subunit alpha

A P E E H P V L L Actin, cytoplasmic 1

S G V S L A A L K Histone H1.2

P V P V L V E D T Mitogen-activated prot.kinase kinase kinase 5

M D N Y S T G Y D Nuclear receptor subfamily 4 group A member 2

I D C L S Q K Q F Activating signal cointegrator 1

T P D E I D H V F Poly [ADP-ribose] polymerase 14

A Y D A T H L V K S-formylglutathione hydrolase

I P L P L I K S Y Cyclin-dependent kinase 2

L P D E I Y H V Y N-terminal Xaa-Pro-Lys N-methyltransferase 1

S N L E N I D F K Adenylosuccinate lyase

I P N E I I H A L Heterogeneous nuclear ribonucleoprot. M

N V I R D A V T Y Histone H4

L P Q E A F E K Y Structural maintenance of chromosomes prot. 3

L K D D E V A Q L L-lactate dehydrogenase B chain

A G L Q F P V G R Histone H2A.Z

Y P V E H P D K F Transitional endoplasmic reticulum ATPase

I D C L S Q K Q F Activating signal cointegrator 1

V E N Q I E K V F Translation factor GUF1, mitochondrial

K L E D G P K F L K Elongation factor 1-alpha 1

G G V V G I K V D K Fructose-bisphosphate aldolase A

G S G T A E V E L K Pyruvate kinase isozymes M1/M2

Q E K I V Q C Q K A Inactive phospholipase C-like prot. 1

M F C Q A A R V D L E3 ubiquitin-prot. ligase TRIM41

H P L S L T S D Q Y Interferon regulatory factor 3

L P S P V T A Q K Y Elongation factor 2

P E L A K S A P A P K Histone H2B type 1-L

P D P A K S A P A P K Histone H2B type

E A K V K F E E R Y K 60S ribosomal prot. L27

P E P A K S A P A P K Histone H2B type 2-E

V L P G V D A L S N I Phosphoglycerate kinase 1

L L K V L S F T H P T Germinal center-associated signaling and motility prot.

N F G I G Q D I Q P K 60S ribosomal prot. L7a

S P V N S S K Q P S Y ATP synthase lipid-binding prot., mitochondrial

A S T S S N S A S S F Zinc finger homeobox prot. 3

V G G T S D V E V N E K 60 kDa heat shock prot., mitochondrial

C D T R P Q L L M R G C Integrin beta-2

A R V I T E E E K N F K 60S ribosomal prot. L13

A L S T G E K G F G Y K Peptidyl-prolyl cis-trans isomerase A

H P I H L G D E Q H S Q Y RING finger prot. 10

K M K I H G V V A F K C E Neurexin-1-alpha

N R Q D P S Q E E E G A A RUN and SH3 domain-containing prot. 1

A G N L G G G V V T I E R 60S ribosomal prot.

F S I V R D P A A L A R S A Galactose-3-O-sulfotransferase 4

S N T A G S Q S Q V E T E A Peptidyl-prolyl cis-trans isomerase FKBP4

N T K G G D A P A A G E D A 40S ribosomal prot. S25

E V S T N T A M I Q T S K T E Keratin, type I cytoskeletal 13

P Y G S R S P F E H S V E H K Chromodomain-helicase-DNA-binding prot. 1

A S G N Y A T V I S H N P E T K 60S ribosomal prot. L8

**Low binding peptides associated with HLA-B*35:01 (origin LCL 721.221 cells)**

Peptide position

1 2 3 4 5 6 7 8 9 10 11 12 13 14 15 16 17 18 19 20 21

Ligands Source

N V A D L H E K Y Proteasome subunit beta type-5

TV F D L V E E Y Nuclear pore complex prot. Nup107

L P Q E A F E K Y Structural maintenance of chromosomes prot. 3

T A T Q L A V N K T-complex prot. 1 subunit eta

H P I R I A D G Y T-complex prot. 1 subunit epsilon

N P I S T V T E L T-complex prot. 1 subunit delta

N A V N L A I K Y WD repeat and HMG-box DNA-binding prot. 1

H A V S P I A K Y PAP-associated domain-containing prot. 5

I G P L G L S P K 60S ribosomal prot. L12

E A I G A V I H Y Neurochondrin

S P I D V V E K Y Kelch-like prot. 12

T P A G V V N K Y 7-dehydrocholesterol reductase

A P E E H P V L L Actin, cytoplasmic 1

L P S P V T A Q K Y Elongation factor 2

I A R D E G G K A F ADP/ATP translocase 2 OS

T P A P V E K S P A Histone H1.5

Y V H D D G R V S Y Glia maturation factor gamma

T V I D E V R T G T Y Tubulin alpha-1A chain

S P V N S S K Q P S Y ATP synthase lipid-binding prot., mitochondrial

P E L A K S A P A P K Histone H2B type 1-L

P D P A K S A P A P K Histone H2B type 1-H

P D P S K S A P A P K Histone H2B type 3-B

K Q T Y S T E P N N L K 60S ribosomal prot. L28

R Q Q P G P S E H I E R Vasodilator-stimulated phosphoprot.

L T S D D V K E Q I Y K 40S ribosomal prot. S13

Q S L P P G L A V K E L 60S ribosomal prot. L38

I A V D G E P L G R V S F Peptidyl-prolyl cis-trans isomerase A

L K A N P F G G A S H A K 40S ribosomal prot. S23

F G F G D S R G G G G N F Heterogeneous nuclear ribonucleoprot.s A2/B1

N A G A V I G K G G K N I K Heterogeneous nuclear ribonucleoprot. K

K I K E K Y I D Q E E L N K Putative heat shock prot. HSP 90-beta 2

A S I K K G E D F V K T L K Malate dehydrogenase, mitochondrial

V A K V T G G A A S K L S K 60S ribosomal prot. L35

D T G K T P V E P E V A I H R 40S ribosomal prot. S20

H G S Y E D A V H S G A L N D T-complex prot. 1 subunit alpha

S Q V I S N A K N T V Q G F K Heat shock 70 kDa prot. 4

N Q Q I T H A N N T V S N F K Heat shock prot. 105 kDa

P E P V K S A P V P K K G S K Histone H2B type 1-M

K L T G K D V N F E F P E F Q L 40S ribosomal prot. S7

H A V S E G T K A V T K Y T S A Histone H2B type 1-K

A S I P F S V V G S N Q L I E A K Septin-2

S A I N E V V T R E Y T I N I H K 60S ribosomal prot. L31

H S G P G P A G F P V P N Q P V Y Phospholipid scramblase 1

S V P T S T V F Y P S D G V A T E K Transketolase

T I L S N Q T V D I P E N V D I T L K 60S ribosomal prot. L9

T A E A G G V T G K G Q D G I G S K A E K Poly [ADP-ribose] polymerase 1

**High binding peptides associated with HLA-B*35:01 (origin LCL 721.221 cells)**

Peptide position

1 2 3 4 5 6 7 8 9 10 11 12 13 14 15 16 17 18 19 20 21

Ligands Source

F P G P S K P F T-cell-specific surface glycoprot. CD28

Y P V E I H E Y L Nuclear nucleic acid-binding prot. C1D

N P D D V F R E F DnaJ homolog subfamily B member 6

N P V N Y G R P Y Ribosome biogenesis prot. TSR3 homolog

Y P V D L G D K F DNA-directed RNA polymerases I, II, and III subunit RPABC3

S A A S E Q H V F Pre-mRNA-splicing factor ATP-dependent RNA helicase PRP16

Q A V A D A V T Y Proteasome subunit beta type-6

H H P A A A A A Y Transcription factor MafA

N V A D L H E K Y Proteasome subunit beta type-5

M P Q E K S P G Y G patch domain-containing prot. 2-like

L P D E I Y H V Y N-terminal Xaa-Pro-Lys N-methyltransferase 1

D P F V D R I G Y Nucleoporin NUP188 homolog

Q P M E V Q E G Y Transcription intermediary factor 1-beta

F P A G K V P A F Elongation factor 1-gamma

M P Q G A P R L Y Phosphatidylinositol 3,4,5-trisphosphate-dependent Rac prot.

T A C A P V S H Y Phosphorylated adapter RNA export prot.

Y P N G V V V H Y Heat shock-related 70 kDa prot. 2

T P I Q S K E A Y Adenylate kinase isoenzyme 4, mitochondrial

M P N G T V Q R F Nucleosome-remodeling factor subunit BPTF

M P E D V K N F Y Tubulin polyglutamylase complex subunit 2

L P D T R S E A Y Cyclin-T1

Y P L D V Q K E F Probable RNA polymerase II nuclear localization prot.

I P L P L I K S Y Cyclin-dependent kinase 2

T P G G T R I I Y Eukaryotic translation initiation factor 4E-binding prot.

Y P D E Y H G E Y Calponin-3

H P T D P L T S F Prot. THEMIS2

N A I S V T T S Y RNA polymerase-associated prot. CTR9 homolog

N A A Q T S V A Y Arginine--tRNA ligase, cytoplasmic

A G L Q F P V G R Histone H2A type 1-A

T A A E T H Y T Y Upstream stimulatory factor 1

L P F D K E T G F SRA stem-loop-interacting RNA-binding prot., mitochondrial

N P L Q K D P Q Y Prot. phosphatase 1 regulatory subunit 7

M P Q G G G Q H Y Prot. SSXT

H P I Q T Q A Q Y La-related prot. 4

F P V I Y D V K Y CCR4-NOT transcription complex subunit 7

Y P N S H T H Y F CCR4-NOT transcription complex subunit 1

F P I P G E P G F Actin-related prot. 2/3 complex subunit 3

F P A K V T A H W Trafficking prot.particle complex subunit 5

T A A G L M H T F Selenide, water dikinase 2

L P Q E A F E K Y Structural maintenance of chromosomes prot.3

F P E E D K K T Y NADH dehydrogenase [ubiquinone] 1 subunit C2

I P N E I I H A L Heterogeneous nuclear ribonucleoprot. M

L P N D G D E K Y Programmed cell death prot. 2-like

I P A A V K L T Y E3 ubiquitin-prot.ligase UBR5

T P A G V V N K Y 7-dehydrocholesterol reductase

D A G P P T H A F E3 ubiquitin-prot. ligase pellino homolog 1

M P A E I V E L H Transcriptional repressor prot. YY1

E A A V A I K A M Eukaryotic translation initiation factor 5A-1

S P I D V V E K Y Kelch-like prot. 12

T A S A V V Q H M Splicing factor 3B subunit 1

T P M F V V K A Y Elongation factor 2

F P N A I E H T L Ubiquitin-like modifier-activating enzyme 1

H A D G T I V R Y Intraflagellar transport prot. 172 homolog

T P A E I R E E F DnaJ homolog subfamily C member 11

A P V E V T H N F Regulator of nonsense transcripts 1

L P P G V H I S Y Integrin beta-7

N A F Y E H A Q T Y Trafficking prot.particle complex subunit 11

Q P W E E I K T S Y Ectonucleoside triphosphate diphosphohydrolase 1

S P A Q E D G K V Y Hematopoietic cell signal transducer

F P A G I Y D T K Y Target of EGR1 prot. 1

F A V D L E H H S Y Exosome component 10

F P V K G L K T G Y 1-phosphatidylinositol bisphos phosphodiest.gamma

N V V K L L G E Q Y Zinc finger prot. ubi-d4

Q A F Q E R L N S Y Nuclear pore complex prot. Nup155

F P M T H G N T G F Poly(rC)-binding prot. 2

M P S Q V V K G G A F EH domain-containing prot.

M P E P Q A P G R Y F Ig mu chain C region, Ig mu heavy chain disease prot.

V P C D S N E A N E M Sperm prot.associated with the nucleus on the X chrom A

H P T D I T S L D Q Y Endoplasmin

L P F P D E T H E R Y Trafficking prot.particle complex subunit 2-like prot.

T P I Q D N V D Q T Y Germinal center-associated signaling and motility prot.

E A F D E L L A S K Y Regulator of G-prot.signaling 2

D A I R S L A S V S Y ATP-dependent RNA helicase DDX50

R P F E E N G A C K Y Zinc finger prot. 36, C3H1 type-like 1

V P E E G G A T H V Y A-kinase-interacting prot. 1

L P F D G S P K I T Y HERV-V_19q13.41 provirus ancestral Env polyprot. 1

N P E N L A T L E R Y Eukaryotic translation initiation factor 3 subunit K

M P V G P D A I L R Y Large proline-rich prot. BAG6

Q V H P D T G I S S K Histone H2B type 1-A

L P Y K A T E N D I Y Heterogeneous nuclear ribonucleoprot. F

Q P L L I I G K G A A Y 2-hydroxyacyl-CoA lyase 1

S L V S K G T L V Q T K Histone H1.1

F P A V G E P N I Q Q Y UDP-N-acetylglu. N-Acetylglu.tranf. prot.

V P F E S E D N Q G I V Y Prot. flightless-1 homolog

M P W G D P N Y R S A N F RING finger prot. 114

H P I S S E E L L S L K Y Prot. C-ets-1

H A F Q P Q S G T V E A M Cirhin

T P W E E S T N D I S H Y Phosphoinositide 3-kinase regulatory subunit 5

T L H P D L G T D K D K E Q W K L-lactate dehydrogenase A chain

L I A P V A E E E A T V P N N K L-lactate dehydrogenase B chain

H A V S E G T K A V T K Y T S A Histone H2B type 1-J

**Low binding peptides associated with HLA-B*35:08 (origin LCL 721.220 cells)**

Peptide position

1 2 3 4 5 6 7 8 9 10 11 12 13 14 15 16 17 18 19 20 21

Ligands Source

R Y R T N T G F Intelectin-2

I D S K C L H F Histone acetyltransferase KAT5

I D D Y E L P L Inactive dipeptidyl peptidase 10

M A D S S P A L LysM and peptidoglycan-binding domain-containing prot. 2

S P S E K E D E Biorientation of chromosomes in cell division prot. 1-like

M A D E A A L A NAD-dependent prot. deacetylase sirtuin-1

M A L A D S A I Insulin receptor substrate 4

V Y S H D G V S L Frataxin, mitochondrial

F P E G S V E L Y 40S ribosomal prot. S3

M S V Q A L Q S L RAD9, HUS1, RAD1-interacting nuclear orphan prot. 1

V P L A F G P P P Structure-specific endonuclease subunit SLX1

A A T L E E G N P Trinucleotide repeat-containing gene 18 prot.

F P D K P I T Q Y Dolichyl-diphosphooligosaccharide--prot. glycosyltransferase

L P A V G E H V F E3 SUMO-prot. ligase CBX4

I P Y Q D R E S Y Zinc finger prot. 106 homolog

F P S S N V H V Y DnaJ homolog subfamily B member 12

M P D E A T P H Y Alpha-mannosidase 2

F P I P G E P G F Actin-related prot. 2/3 complex subunit 3

L P S E I E V K Y Anaphase-promoting complex subunit 7

L P N D G D E K Y Programmed cell death prot. 2-like

P G H P G P S E P Microtubule-associated serine/threonine-prot.kinase 4

H P D A P M S Q V Y Mortality factor 4-like prot. 1

G Q K L E G L L R Q Leucine-rich repeat-containing prot. 16C

L K S T V S S L L Q BPI fold-containing family A member 2

Y K C V S C T K T F Zinc finger prot. 668

F P D V P D K E N F Prot. CASC5

F P D T G S D H S Y THAP domain-containing prot. 11

A F G G G E R V S L Putative uncharacterized prot. SPANXA2-OT1

I P A S N T A D E E Y Inositol 1,4,5-trisphosphate receptor type 2

F P Q E E A I I D K Y All-trans-retinol 13,14-reductase

Y M I F D P N N P L M Zinc finger homeobox prot. 4

V S E T A S G S V T Q P K CD2-associated prot.

V G N G S A L P T N D N S Y Corneodesmosin

**High binding peptides associated with HLA-B*35:08 (origin LCL 721.220 cells)**

Peptide position

1 2 3 4 5 6 7 8 9 10 11 12 13 14 15 16 17 18 19 20 21

Ligands Source

P A Q G A K Y R Annexin A6

Q L Y G G R A A Lipocalin-like 1 prot.

S K Q A E E E F Threonine--tRNA ligase, cytoplasmic

A L D R I V E Y Prot. lunapark

P A K S A P A P K Histone H2B type 1-H

L P Q E A F E K Y Structural maintenance of chromosomes prot. 3

F P D K P I T Q Y Dolichyl-diphospholip--prot.glycosyltransferase

V Y P A D V V L F Uridine-cytidine kinase 1

H P D K K I V A Y Ataxin-10

T P D E I D H V F Poly [ADP-ribose] polymerase 1

H P N D D D V H F 5'-3' exoribonuclease 1

P G P P P P G P H Prot.transportprot. Sec24D

L P D T R S E A Y Cyclin-T1

L V P A T A E P P Telomere length regulation prot. TEL2 homolog

P H L G P P V P P POU domain, class 3, transcription factor 3

P P G P P P P Q P Prot. phosphatase 1 regulatory subunit 28

L P D T L K V T Y Integrin beta-2

A D R D R P S V P Prot. KIAA0284

L S D G V A V L K 60 KDa heat shock protein, mitochondrial

A P P P P P P G H Zinc finger prot. ZFPM1

F P I P D L Q K Y Insulin-degrading enzyme

Y P T E D Y K V Y Trafficking prot.particle complex subunit 2-like prot.

A G L Q F P V G R Histone H2A type 1-A

L P N D G D E K Y Programmed cell death prot. 2-like

L P D E K V E L F Pericentriolar material 1 prot.

T G S R G E L M L E Kinesin-like prot.KIF12

Y A Q D E H L I T F U5 small nuclear ribonucleoprot. 200 kDa helicase

Y P D E N G F D A F Nardilysin

F P E T T S P H E Y Neuroblastoma-amplified sequence

L A G E S E S N L R Transitional endoplasmic reticulum ATPase

H P E S S L S S E E Receptor-type tyrosine-prot. phosphatase

P E P A K S A P A P K Histone H2B type 1-C/E/F/G/I

P E P V K S A P V P K Histone H2B type 1-M

T V I D E V R T G T Y Tubulin alpha-1B chain

P E P T K S A P A P K Histone H2B type 1-D

L P Y R A T E N D I Y Heterogeneous nuclear ribonucleoprot.

F P Q E E A I I D K Y ll-trans-retinol 13,14-reductase

D S Y V G D E A Q S K Actin, aortic smooth muscle

D S Y V G D E A Q S K Actin, cytoplasmic 1

A P A V T Q H A P Y F K Thioredoxin-dependent peroxide reductase

A L S T G E K G F G Y K Peptidyl-prolyl cis-trans isomerase A

S V Q A T T E N K E L K Delta(3,5)-Delta(2,4)-dienoyl-CoA isomerase, mitochondrial

P N S S I F L T D T A K Tryptophan--tRNA ligase, cytoplasmic

N E D T S H A A T T I P E Collagen alpha-1(XVIII) chain

A A A A E I D E E P V S K Nascent polypeptide-associated complex subunit alpha

S N T A G S Q S Q V E T E A Peptidyl-prolyl cis-trans isomerase FKBP4

H G S Y E D A V H S G A L N D T-complex prot. 1 subunit alpha

A Q A A A P A S V P A Q A P K 60S ribosomal prot.

R L S A K P A P P K P E P K P K Non-histone chromosomal prot. HMG-17

H A V S E G T K A V T K Y T S A Histone H2B type 1-J

A Y V R L A P D Y D A L D V A N K 60S ribosomal prot. L23a

P P A E N S S A P E A E Q G G A E Nuclease-sensitive element-binding prot. 1

A S S E G G T A A G A G L D S L H K Actin-related prot. 2/3 complex subunit 1B

**Low binding peptides associated with HLA-B*35:08 (origin LCL 721.221 cells)**

Peptide position

1 2 3 4 5 6 7 8 9 10 11 12 13 14 15 16 17 18 19 20 21

Ligands Source

F P R C E F L I Baculoviral IAP repeat-containing prot. 2

M V F S E T V I Dickkopf-related prot. 3

L P D A H S D Y RNA-binding prot. 14

L S L A E A S R Growth/differentiation factor 15

F P N A I E H T L Ubiquitin-like modifier-activating enzyme 1

T A D V V K V A Y Nuclear pore membrane glycoprot. 210

F P S I Y D V K Y CCR4-NOT transcription complex subunit 8

H P I S S E E L L Prot. C-ets-1

S P Q E K E A L Y ADP-ribosylation factor-binding prot. GGA2

H P D P I V I N H SWI/SNF-related actin-dependent regulator

Y A D P V N A H Y Polypyrimidine tract-binding prot. 3

L P F D K E T G F SRA stem-loop-interacting RNA-binding prot., mitochondrial

N V I R D A V T Y Histone H4

L P S E I E V K Y Anaphase-promoting complex subunit 7

H P H L V A E A Y Speckle-type POZ prot.

F P D K P I T Q Y Dolichyl-diphosphooligosac.glycosyltraf. 48 kDa subunit

I G P L G L S P K 60S ribosomal prot. L12

A A L L K A S P K 60S ribosomal prot. L14

F P A E K E S E W Calcineurin-like phosphoesterase domain-containing prot.

H P N D D D V H F 5'-3' exoribonuclease 1

A V D S Q I L P K 60S ribosomal prot. L6

L P D E K V E L F Pericentriolar material 1 prot.

H A D S I L E K Y Inositol 1,4,5-trisphosphate receptor type 1

H A D G T I V R Y Intraflagellar transport prot. 172 homolog

V I D K E S E V Y PDZ and LIM domain prot. 1

N P D E A E K A L RNA-binding prot.with serine-rich domain 1

Y P D E Y H G E Y Calponin-3

A Y D A T H L V K S-formylglutathione hydrolase

L P Q E A F E K Y Structural maintenance of chromosomes prot. 3

F P E I D L E K Y Dihydrofolate reductase

L P N D G D E K Y Programmed cell death prot. 2-like

A P E E H P V L L Actin, cytoplasmic 1

N A Q R Q D I A F Annexin A2

T A T Q L A V N K T-complex prot. 1 subunit eta

K L E D G P K F L Elongation factor 1-alpha 1

T L A A I Q G L L K Spectrin alpha chain, non-erythrocytic 1

L Q S S R A F T N S Deoxyribonuclease gamma

L V D I E K A I A H Alpha-soluble NSF attachment prot.

T P I E E R G D L F Zinc finger prot. 1 homolog

F P I S E E T I K L Nucleolar RNA helicase 2

R P I E D D Q E V Y FYN-binding prot.

H P D E K S I I T Y Spectrin beta chain, non-erythrocytic 1

L P E V E V P Q H L Nucleobindin-1

H I A N V E R V P F Gelsolin

H P S D I E V D L L Beta-2-microglobulin

P E P V K S A P V P K Histone H2B type 1

H A T V A T E N E V F Gamma-interferon-inducible prot. 16

L P Y K A T E N D I Y Heterogeneous nuclear ribonucleoprot. F

L P Y R A T E N D I Y Heterogeneous nuclear ribonucleoprot. H

H P E N P K A V E T F DnaJ homolog subfamily C member 12

S I S E G D D K I E Y rRNA 2'-O-methyltransferase fibrillarin

T V I D E V R T G T Y Tubulin alpha-1A chain

F P I P A A E V D R L Nuclear receptor 2C2-associated prot.

H A Q H E G E S V S Y Nuclear receptor coactivator 2

F P Q E E A I I D K Y All-trans-retinol 13,14-reductase

V P E E G G A T H V Y A-kinase-interacting prot. 1

L K D D E V A Q L K K L-lactate dehydrogenase B chain

N I D D G T S D R P Y 60S ribosomal prot. L27

A L S T G E K G F G Y K Peptidyl-prolyl cis-trans isomerase A

A T G P P V S E L I T K Histone H1.5

V K A E P A K I E A F R Phosphoglycerate kinase 1

F G K K T G E G F Y K Y Hydroxyacyl-coenzyme A dehydrogenase, mitochondrial

A R V I T E E E K N F K 60S ribosomal prot. L13

F A Q I N Q G E S I T H Adenylyl cyclase-associated prot. 1

D F S E L E P D K F Q N K Glycogen phosphorylase, liver form

S A D T L W G I Q K E L Q F L-lactate dehydrogenase A chain

Q G S V Q K V Y N G L Q G Y DnaJ homolog subfamily B member 11

S P D D P S R Y I S P D Q L Alpha-enolase

E A M K A Q M A E K E A I L Q FYVE and coiled-coil domain-containing prot.1

Y G N Q G G G Y G G G Y D N Y Heterogeneous nuclear ribonucleoprot.s A2/B1

H A V S E G T K A V T K Y T S A Histone H2B type 1-J

H A V S E G T K A V T K Y T S S Histone H2B type 1-L

H A V S E G T K A V T K Y T S S Histone H2B type 1-H

K G D D Q S R Q G G A P D A G Q E T-complex prot. 1 subunit gamma

G A D F L V T E V E N G G S L G S K Pyruvate kinase isozymes M1/M2

S V P T S T V F Y P S D G V A T E K Transketolase

**High binding peptides associated with HLA-B*35:08 (origin LCL 721.221 cells)**

Peptide position

1 2 3 4 5 6 7 8 9 10 11 12 13 14 15 16 17 18 19 20 21

Ligands Source

D E G W F L I L Activating signal cointegrator 1 complex subunit 3

F P E D E G I H Y B-cell receptor CD22

F P Q E G M H Q M Probable global transcription activator SNF2L2

I A D K P M T Q Y Survival of motor neuron-related-splicing factor 30

M P A A T D N R Y Aminoacylase-1

L P Q K G D V E M DNA (cytosine-5)-methyltransferase 1

S P Q E K E A L Y ADP-ribosylation factor-binding prot. GGA2

F P Q Y P D K E L CCR4-NOT transcription complex subunit 1

L P F D K E T G F SRA stem-loop-interacting RNA-binding prot., mitochondrial

F P E P E H S S F Transmembrane and coiled-coil domain-containing prot. 4

T A A A P M I G Y Probable ATP-dependent RNA helicase DDX5

F P D V E K A E W Extended synaptotagmin-1

H P D V A K V S F 4-trimethylaminobutyraldehyde dehydrogenase

Y P D S K D L T M Dual specificity tyrosine-phosphorylation-regulated kinase 4

H P N D D D V H F 5'-3' exoribonuclease 1

M P E K N E H S Y Pentatricopeptide repeat-containing prot. 3, mitochondrial

F P L T T E S A M 60S ribosomal prot. L23a

T A D P S H Q T M SPATS2-like prot.

Y P N E E K D A W Disintegrin metalloprot.ase domain-containing prot. 17

M P M V T D N K M Serine/threonine-prot. kinase ATR

M A D Y S D P S Y Cell division cycle 5-like prot.

Q A I E D M V G Y 26S proteasome non-ATPase regulatory subunit 1

M P I G P D V S L Spermatogenesis-associated prot. 5-like prot. 1

F P D K P I T Q Y Dolichyl-diphospho--prot.glycosyltransferase

M P D V V V R S F Mediator of RNA polymerase II transcription subunit 27

H A D G T I V R Y Intraflagellar transport prot.172 homolog

F P D E T H E R Y Glycosylphosphatidylinositol anchor attachment 1 prot.

F P E E L T Q T F Elongation factor 1-gamma

H A D K P V A T Y ATP-binding cassette sub-family A member 13

Y A D P V N A H Y Polypyrimidine tract-binding prot. 3

L P D E K V E L F Pericentriolar material 1 prot.

L P D T L K V T Y Integrin beta-2

E A A P H D I G Y N-acetyl-D-glucosamine kinase

Q A V A D A V T Y Proteasome subunit beta type-6

F P E I D L E K Y Dihydrofolate reductase

F P M P G F D E H Serine hydroxymethyltransferase, mitochondrial

M P D E A T P H Y Alpha-mannosidase 2

Y P N G V V V H Y Vam6/Vps39-like prot.

M A A D D V E E Y Programmed cell death prot. 10

N V E E A D A A M Heterogeneous nuclear ribonucleoprot. A0

N V I R D A V T Y Histone H4

M P M G P D Q K Y Nuclear receptor coactivator 3

T P E E K E Q V Y Homologous-pairing prot. 2 homolog

L P S E I E V K Y Anaphase-promoting complex subunit 7

L P D T R S E A Y Cyclin-T1

M T A D R D P V Y DNA repair prot. XRCC4

F P A E K E S E W Calcineurin-like phosphoesterase domain-containing prot. 1

F P S S N V H V Y DnaJ homolog subfamily B member 12

F P N A I E H T L Ubiquitin-like modifier-activating enzyme 1

M A D Y P D Y K Y Transcription factor SOX-4

L P V A K D V S Y UTP--glucose-1-phosphate uridylyltransferase

N A D P Q A V T M Melanoma-associated antigen D2

N A A Q T S V A Y Arginine--tRNA ligase, cytoplasmic

L P D E I Y H V Y N-terminal Xaa-Pro-Lys N-methyltransferase 1

F P E E D K K T Y NADH dehydrogenase [ubiquinone] 1 subunit C2

L P N D G D E K Y Programmed cell death prot. 2-like

F P M T M D E K Y Cullin-3

T P D E I D H V F Poly [ADP-ribose] polymerase 14

T A D V V K V A Y Nuclear pore membrane glycoprot. 210

F P I P G E P G F Actin-related prot. 2/3 complex subunit 3

M P L E E G D T F Splicing factor 3B subunit 3

H P D A P M S Q V Y Mortality factor 4-like prot.

N P D D I T N E E Y Heat shock prot. HSP 90-alpha

M P N S A S R D E F Dedicator of cytokinesis prot. 11

F P E E F D K T S F N-acetylglucosamine-1-phosphotransferase subunits alpha/beta

N P D D I T Q E E Y Heat shock prot. HSP

Y A Q D E H L I T F U5 small nuclear ribonucleoprot. 200 kDa helicase

H P D G P E G Q A Y Lysine--tRNA ligase

M P V D P N E P T Y Inhibitor of growth prot.4

F P D V P D K E N F Prot. CASC5

R P I E D D Q E V Y FYN-binding prot.

I P I A G R D I T Y Actin-related prot. 3B

L P I E N D T Y K Y Syntaxin-binding prot. 3

M P P Q D A E I G Y Dual specificity prot.kinase TTK

F P E T T S P H E Y Neuroblastoma-amplified sequence

M P G E G E V V R Y Prot. RRNAD1

Y P D P S K Q K P M Tyrosine--tRNA ligase, cytoplasmic

L P I E N D V Y K Y Syntaxin-binding prot. 1

Y P E G F E I H S M Long-chain-fatty-acid--CoA ligase 4

F P D T G S D H S Y THAP domain-containing prot. 11

M P I K N T N Q D I Y Peptidyl-prolyl cis-trans isomerase-like 4

H P D T G I S S K A M Histone H2B type 1-A

N A E P A R P D I T Y Coiled-coil-helix domain-containing prot. 2, mitochondrial

L P Y R A T E N D I Y Heterogeneous nuclear ribonucleoprot. H

F P Q E E A I I D K Y All-trans-retinol 13,14-reductase

H P A E D T E G T E F Centromere prot. F

H P E D S E Y E A E M Ubiquitin carboxyl-terminal hydrolase 16

S I S E G D D K I E Y rRNA 2'-O-methyltransferase fibrillarin

V P E E G G A T H V Y A-kinase-interacting prot.

L P D A H S D Y A R Y RNA-binding prot. 14

M P S K E D A I E H F Poly [ADP-ribose] polymerase 1

M P S Q V V K G G A F EH domain-containing prot. 1

N P D E H S D S E M Y B-cell linker prot.

T P I Q D N V D Q T Y Germinal center-associated signaling and motility prot.

E P D S S T D M E Q Y Apoptosis-enhancing nuclease

L P Y K A T E N D I Y Heterogeneous nuclear ribonucleoprot. F

N A E D A D G K D V F C1GALT1-specific chaperone 1

F P Q S E L G R A E A Y DNA-directed RNA polymerase I subunit

L P N D E T R V N A T M Ubiquitin conjugation factor E4

**Low binding peptides associated with HLA-B*35:62 (origin LCL 721.220cells)**

Peptide position

1 2 3 4 5 6 7 8 9 10 11 12 13 14 15 16 17 18 19 20 21

Ligands Source

F P Q V I K S K Fructose-bisphosphate aldolase A

M P S L P S Y K Transketolase

Q A I D K M Q M COP9 signalosome complex subunit 3

D Q N N K L S K Myosin-11

L G L L M G T G Anaphase-promoting complex subunit 2

S P G S V V F R Renin receptor

I A P G D E T A Uncharacterized family 31 glucosidase KIAA1161

D V S D L L H Q Y Proteasome subunit beta type-8

D S S T V T H L F Proteasome subunit alpha type-6

A Y D A T H L V K S-formylglutathione hydrolase

T A A D I F K Q Y Cohesin subunit SA-2

A P V K K L V V K 60S ribosomal prot. L22

Y I D Q E E L N K Heat shock prot. HSP

A A N P H S F V F Suppressor of SWI4 1 homolog

S G S D I V K L Y Erythroid differentiation-related factor 1

E A D K T I K V Y Pleiotropic regulator 1

D I D T R S E F Y Actin-related prot. 2

K Q L N L L F A K Zinc finger SWIM domain-containing prot. 6

S P M D R N S D E Y CCAAT/enhancer-binding prot.gamma

G V R G A S K E V V Kelch repeat and BTB domain-containing prot. 13

K F D Q L L A E E K Myosin-9

T P M E D V L H S F Aspartate aminotransferase, mitochondrial

S V S D N D I R K Y Transitional endoplasmic reticulum ATPase

R A V D L I Q K H K Flap endonuclease 1

S V E T L K E M I K Pyruvate kinase isozymes M1/M2

E A A V E D L H H Y Phosphatidylserine decarboxylase proenzyme

K F D Q L L A E E K Myosin-14

S K K G I E E S L R Hydroxyacyl-coenzyme A dehydrogenase, mitochondrial

D V Y D D G K H V Y Ribosomal prot. S6 kinase alpha-1

L L D V V H P A A K T-complex prot. 1 subunit eta

L A L L S L S G L E Beta-2-microglobulin

T F H T I G F C P Y Zinc finger prot. 36, C3H1 type-like 2

D A L D D Y E H H Y Zinc finger prot. 511

H P L V L Q E C V S D Lysine-specific demethylase 4A

N T V G Q N E L K I T Centrosome and spindle pole-associated prot. 1

S A I N E V V T R E Y 60S ribosomal prot. L31

V L M T Q Q P R P V L H/ACA ribonucleoprot. complex subunit 3

E I T A L A P S T M K Actin, cytoplasmic 1

A E L L D N E K P A A Calcyclin-binding prot.

F Y N Q V S T P L L R Inter-alpha-trypsin inhibitor heavy chain H2

P M F I V N T N V P R Macrophage migration inhibitory factor

A A E I D E E P V S K Nascent polypeptide-associated complex subunit alpha

P E P V K S A P V P K Histone H2B type 1-M

A M E A V A A Q G K A Phosphoglycerate mutase 1

G P S S V E D I K A K Nucleophosmin

T A I I E E Q P K N Y Prot. Farnesyl/geranyl transferase type-1subunit alpha

S K P V F S E S L S D Mitochondrial import inner membrane translocase subunit Tim8

D A L K D S D L L H W Anaphase-promoting complex subunit 5

N A S E D E I K K A Y DnaJ homolog subfamily C member 7

H I T D C R L T N G S R Ribonuclease pancreatic

A D L A E T R P D L K N Layilin

D N I Q G I T K P A I R Histone H4

A L R Y P M A V G L N K 60S ribosomal prot. L36

L T S D D V K E Q I Y K 40S ribosomal prot. S13

A L S T G E K G F G Y K Peptidyl-prolyl cis-trans isomerase A

E P L P S E V T S N H F DNA polymerase theta

D A L E T L G F L N H Y Heterogeneous nuclear ribonucleoprot. L

R Q Q P G P S E H I E R Vasodilator-stimulated phosphoprot.

S V Q A T T E N K E L K Delta(3,5)-Delta(2,4)-dienoyl-CoA isomerase, mitochondrial

P Q E Q A D A A K F M A Brain-specific angiogenesis inhibitor 3

T F N P G A G L P T D K U2 small nuclear ribonucleoprot. A'

G T V S V A D T K G V K Low-density lipoprot.receptor

A L G Q N P T N A E V L K Myosin light polypeptide 6

H A Q G E K T A G I N V R T-complex prot. 1 subunit delta

S V L I S L K Q A P L V H Clathrin light chain A

A T D F V A D R A G T F K Isocitrate dehydrogenase [NADP], mitochondrial

N G V M P S H F S R G S K 40S ribosomal prot. S19

L K L Q E E Q F V N A V E Peptidase M20 domain-containing prot. 2

V A K V S Q G V E D G P D SUMO-activating enzyme subunit 1

N S V S Q I S V L S G G K Actin-related prot. 2/3 complex subunit 1B

T V V N K D V F R D P A L 60S ribosomal prot. L27

S V S L T G A P E S V Q K Far upstream element-binding prot. 2

A Q P A Q P A D E P A E K Proteasome subunit alpha type-1

K F K Y P Q A P V I M G N Aldehyde oxidase

Q A G W T A C G A V D M N Leucine carboxyl methyltransferase 2

D S D G D E D Q G E G E A Leucine-rich repeat and guanylate kin. domain prot.

L G G S A V I S L E G K P L Cofilin-1

V P S G Q N A D V Q K T D N N-alpha-acetyltransferase 50

G A V D G G L S I P H S T K 60S ribosomal prot. L5

G M G T V E G G D Q S N P K Treacle prot.

A A V A G K K P V V G K K G 60S ribosomal prot. L4

I V S G K D Y N V T A N S K L-lactate dehydrogenase A chain

I V A D K D Y S V T A N S K L-lactate dehydrogenase B chain

A V R V F E F G G P E V L K Quinone oxidoreductase

I F V G G L S P D T P E E K Heterogeneous nuclear ribonucleoprot. D0

V A K V T G G A A S K L S K 60S ribosomal prot. L35

F S E S R A D E V A P A K K ATP-citrate synthase

K L G G F S D D L H K T P G Outer dense fiber prot. 3

T V T P A K A V T T P G K K Nucleolin

I F F T D L D S F L I T S M A Olfactory receptor 1J2

H G S Y E D A V H S G A L N D T-complex prot. 1 subunit alpha

A Q A A A P A S V P A Q A P K 60S ribosomal prot. L29

L G D V Y V N D A F G T A H R Phosphoglycerate kinase 2

S Q V I S N A K N T V Q G F K Heat shock 70 kDa prot. 4

A S G N Y A T V I S H N P E T 60S ribosomal prot. L8

L V G S Q K E P S E V P T P K High mobility group prot. HMG-I/HMG-Y

E A M K A Q M A E K E A I L Q FYVE and coiled-coil domain-containing prot.1

S E A V A D R E D D P N F F K Glutamate dehydrogenase 1, mitochondrial

I R L T I D T T Q T I S E D T D Prot.FAM188B

R P G C Q G G S E R G S I P L L Uncharacterized prot. C2orf71

G G K P E P P A M P Q P V P T A 40S ribosomal prot. S3

A Y V R L A P D Y D A L D V A N 60S ribosomal prot. L23a

H A V S E G T K A V T K Y T S A Histone H2B type 1-K

P D P A K S A P A P K K G S K K Histone H2B type 1-H

S T A T D I T G P I I L Q T Y R Putative neutrophil cytosol factor 1C

A Q L G G P E A A K S D E T A A Heat shock prot. beta-1

K L T G K D V N F E F P E F Q L 40S ribosomal prot. S7

K R K D T T S D K D D S L G S Q Nuclear autoantigen Sp-100

G A P G P G P A D A S K V V A K Filamin-A

N D Q A N Y S L N T D D P L I F K Adenosine deaminase

H A V S E G T K A V T K Y T S S K Histone H2B type 1-C/E/F/G/I

K E E S E E S D D D M G F G L F D 60S acidic ribosomal prot. P1

A T S N V F A M F D Q S Q I Q E F K Myosin regulatory light chain 12A

A A N D A G Y F N D E M A P I E V K 3-ketoacyl-CoA thiolase, mitochondrial

F R I L C T T E D G L L R F V S P V WD repeat-containing prot. 87

A G L C T E G A L L L L E M L K A T M S WD repeat- and FYVE domain-containing prot. 4

E I L L K K C T N I L N S N G E L R G F Plasma membrane calcium-transporting ATPase 3

**High binding peptides associated with HLA-B*35:62 (origin LCL 721.220 cells)**

Peptide position

1 2 3 4 5 6 7 8 9 10 11 12 13 14 15 16 17 18 19 20 21

Ligands Source

Y V R D I S A Y Serine/threonine-prot.kinase Nek10

M K E V T R T W Trimeric intracellular cation channel type B

G H T H D G G Y Manganese-dependent ADP-ribose/CDP-alcohol diphosphatase

N P N G G F R M Y Plasma membrane calcium-transporting ATPase 4

F P Q D Q I R L W Ubiquitin carboxyl-terminal hydrolase 7

F P N A L V T K L Prot. fem-1 homolog B

T P S P S P H A W Ninein

S P D A T I R I W Pre-mRNA-processing factor 19

T P A S A G H V W Transcription factor SOX-9

A A A D S I K I W WD repeat-containing prot. 3

D P S G T Y H A W Proteasome subunit alpha type-7-like

F P A K V T A H W Trafficking prot.particle complex subunit 5

A V S A V V H E Y Sterol O-acyltransferase 1

T A A D I F K Q Y Cohesin subunit SA-2

T P S A V F R V W Aladin

D T S D I V H I W Centromere prot. U

H V I L G T Q Q F Eukaryotic translation initiation factor 3

D V N S L L K Y F NADH dehyd.[ubiquinone] 1 alpha subcomplex assembly factor 4

D P V D I Y K S W Ras GTPase-activating-like prot. IQGAP1

E G N P D T H S W Ubiquitin-associated prot. 2-like

D V S D L L H Q Y Proteasome subunit beta type-8

E L F E G V Q K W Prot. regulator of cytokinesis 1

S A M D V A K A Y Ankyrin repeat and EF-hand domain-containing prot. 1

T P M F V V K A Y Elongation factor 2

D V T E S N A R W Parkinson disease 7 domain-containing prot. 1

N V S K V S T T W F-box only prot. 5

L P S E L E R S Y Cullin-1

D A G P P T H A F E3 ubiquitin-prot. ligase pellino homolog 2

E A D G G L K S W F-actin-capping prot.subunit alpha-1

D V T G V V R Q W Transforming growth factor beta-1

D V D G V I K V W WD repeat-containing prot. 91

S A S E V L K E W Spermatogenesis-associated serine-rich prot. 2

D I S E K E Q R W Hematopoietic lineage cell-specific prot.

D A N P L K T L W Leucine-rich repeat-containing prot. 33

D A I G P R E Q W Prot. FRG1B

N A T L S V H Q L Tubulin beta-6 chain

H P Q D G R S A W Sterol regulatory element-bind.prot.cleavage-activ.prot.

M P A D T N K A F ER membrane prot.complex subunit 3

Q A T P T F H Q W Ena/VASP-like prot.

Q P S Q A Q R M Y Tetratricopeptide repeat prot. 19

H P I Q T Q A Q Y La-related prot. 4

M A I E A Q Q K F Histone acetyltransferase type B catalytic subunit

H A S D R I I A L Transketolase

A P D N I I K F Y GON-4-like prot.

D A I N D A N L L Farnesyl pyrophosphate synthase

G V A E S I H L W WD repeat-containing prot. 18

N P Q P M T P P W Prot. Asterix

E V S F V I H N L Selenide, water dikinase 2

E A I K Q A S E W Poly [ADP-ribose] polymerase 4

E A M R L G P G W Prot. syndesmos

Y P N V N I H N F Spectrin beta chain, non-erythrocytic 1

N V I D S K E L W Mdm2-binding prot.

T P G E S L H G Y Transmembrane prot. 214

E A T A L V H T L Putative GTP-binding prot. 6

S A S P H A A Y Y Malignant fibrous histiocytoma-amplified sequence 1

E P K A L V S E W DNA damage-binding prot. 1

D Q M I S R I E Y Casein kinase I isoform epsilon

Y P F S S E Q K W Calcium-transporting ATPase type 2C member 1

H V S T V N P N Y Homeobox prot. Hox-C4

D V M A H V H T F Adenylosuccinate lyase

T A A D V V K Q W RalA-binding prot. 1

L A N D V A E Q W Ubiquitin-conjugating enzyme E2 N

S A S P T S P D Y SAM and SH3 domain-containing prot. 3

Q A A E R P Q E W NF-kappa-B inhibitor alpha

E A I Y E T H T W Plasma alpha-L-fucosidase

A A N P I S G H Y DNA replication licensing factor MCM6

S N S A S P H R W E3 SUMO-prot. ligase RanBP2

T A N G T P K V Y Pro-interleukin-16

A A N P H S F V F Suppressor of SWI4 1 homolog

N P A A S S N H W Dual specificity prot. kinase TTK

A A Q D F F Q R W AP-2 complex subunit alpha-1

E P S E S N M R W Mediator of RNA polymerase II transcription subunit 12

D P N G A G S E W KRAB domain-containing prot. ZNF747

E P L P R N G D Q W Wee1-like prot. kinase

N A M D N T V R V W U5 small nuclear ribonucleoprot. 40 kDa prot.

S P M D R N S D E Y CCAAT/enhancer-binding prot. gamma

F G T A G L R S A M lucose 1,6-bisphosphate synthase

E A A T A Q R E E W Heterogeneous nuclear ribonucleoprot.

D A M I V N K A S W DNA-directed RNA polymerase I subunit RPA2

E A Q D A G Y R M Y Ser/thr.-prot. Phosph. 2B catalytic subunit beta isoform

E V S P A T E R Q W RNA-binding prot. 12

T P M E D V L H S F Tight junction prot. ZO-2

A A S G N A V R M W Kinesin-like prot. KIF21A

N A I Q D S L T R W Valine--tRNA ligase

T A Q S D N K I T W V-type proton ATPase catalytic subunit A

H A A D P I I T R W ADP-ribose pyrophosphatase, mitochondrial

E A A V E D L H H Y Phosphatidylserine decarboxylase proenzyme

A H L Y F Q A H G S Pleckstrin homology domain-containing family N member 1

E A Q A V T T S K W U2 snRNP-associated SURP motif-containing prot.

G F G D Y V A G A D Potassium channel subfamily K member 4

D A Q Q S L Q S F W Nuclear transcription factor Y subunit gamma

A P S P Q D R P S F Tyrosine-prot. kinase JAK3

D V M A P D V E V S Prot. AHNAK2

S P I T S S P P K W Prot. lin-52 homolog

F L S P E Q H A C S Fibroblast growth factor-binding prot. 3

A A A G G G R S P E PH domain leucine-rich repeat-containing prot. phosphatase

P D P G G K S Q D A Transmembrane and coiled-coil domains prot. 1

V C P E C A K I S V Ubiquitin carboxyl-terminal hydrolase 15

P A G N C T D E E G I Mucin-5B

S A S D D G T V R I W WD repeat-containing prot. 26

T A A A D F T A K V W Serine-threonine kinase receptor-associated prot.

E A I S D S L L R K Y Putative ATP-dependent RNA helicase DHX33

D Q M P Q G A P R L Y Phosphatidylinositol 3,4,5-trisphosphate-dep. Rac exch.prot.

T A I I E E Q P K N Y Prot. farnesyl/geranylgeranyltransferasesubunit alpha

S P A P A G E R R I Y Zinc finger prot. 511

N V I K E A E A Q L W Uncharacterized prot. C21orf59

T P L E D V G K Q V W Methyltransferase-like prot. 22

G E I E S P A S S F H Integrin beta-5

D A M M A N A A Q K F Asparagine synthetase [glutamine-hydrolyzing]

D V L S D P Q E R A W DnaJ homolog subfamily C member 21

T A S E D G S V R L W Telomerase prot. component 1

H A S D V L E T S G W Speckle-type POZ prot.

S A S S V T V T R S Y Prelamin-A/C

T A S A D G T I K L W Transducin beta-like prot.3

G E I G E K G Q K G E Collagen alpha-1(IV) chain

S A A A D S A V R L W Elongator complex prot. 2

S A T N D A S L H V W WD repeat-containing prot. 73

S A A A D E T L R L W Cell division cycle prot. 20 homolog

A P S G Y V A G H G W Paired box prot. Pax-9

S E M E V Q D A E L K Proliferation-associated prot. 2G4

T C L E P T N V V A W Tetratricopeptide repeat prot. 18

E A L G D N V K Q Y W Transcriptional adapter 1

T A S D D A T I K A W F-box/WD repeat-containing prot. 5

Y P M E N G I V R N W Actin-related prot. 2

S A I N E V V T R E Y 60S ribosomal prot. L31

A P T A E A P P P S V Probable ATP-dependent RNA helicase DDX17

T P I Q D N V D Q T Y Germinal center-associated signaling and motility prot.

D A L D V D D Y R F V JmjC domain-containing prot.4

T V D G P S G K L W R Glyceraldehyde-3-phosphate dehydrogenase

S R K D P S G A S N P S Hepatocyte nuclear factor 3-alpha

T P L P E P D L T R L Y Prot. asteroid homolog 1

S N S G A G V L P S P A Mediator of RNA polymerase II transcription subunit 13-like

Y A M D N S G E H V T W Cation-independent mannose-6-phosphate receptor

M G G K V P P A T Q K A Hsc70-interacting prot.

V G G T S D V E V N E K 60 kDa heat shock prot., mitochondrial

G M K A A L Q V S M N D Anthrax toxin receptor 1

T A S G V D V G G Q H E W Transcription elongation factor SPT5

M L P H P T P I S T E A N Neurocan core prot.

L D L G P S M L D A V L G V M Cdc42 effector prot. 5

**Low binding peptides associated with HLA-B*35:62 (origin LCL 721.221cells)**

Peptide position

1 2 3 4 5 6 7 8 9 10 11 12 13 14 15 16 17 181 19 20 21

Ligands Source

G A L A L E E K Myosin-9

P V G A A N F R Alpha-enolase

R L A S S V L R 60S ribosomal prot. L19

R V I G S G C N L-lactate dehydrogenase B

A P I I A V T R Pyruvate kinase isozymes M1/M2

S P L H E R I Y Proteasome subunit beta type-9

F E M L S F W L Unconventional myosin-Vc

E S S E S L P K Activating transcription factor 7-interacting prot. 1

Y L T D P V L F Latrophilin-2

V P N P D P V T M Rac GTPase-activating prot. 1

F P N A I E H T L Ubiquitin-like modifier-activating enzyme 1

I G P L G L S P K 60S ribosomal prot. L12

L P S E I E V K Y Anaphase-promoting complex subunit 7

M P A E I V E L H Transcriptional repressor prot. YY1

S P I D V V E K Y Kelch-like prot. 12

T P A G V V N K Y 7-dehydrocholesterol reductase

N A M N M Q E T Y Sorting nexin-4

F P V I Y D V K Y CCR4-NOT transcription complex subunit 7

I P A A V K L T Y E3 ubiquitin-prot. ligase UBR5

S N L E N I D F K Adenylosuccinate lyase

A I A T G G A V F 60 kDa heat shock prot., mitochondrial

H P N D D D V H F 5'-3' exoribonuclease 1

N P F E K G D L Y DnaJ homolog subfamily A member 2

M P N S P A P H F Absent in melanoma 1 prot.

N A I K E S Y D Y Polycomb prot. EED

L P P G V H I S Y Integrin beta-7

I P N E I I H A L Heterogeneous nuclear ribonucleoprot. M

Y P V D L G D K F DNA-directed RNA polymerases I, II, and III subunit RPABC3

M P M G P D Q K Y Nuclear receptor coactivator 3

M A Y G H I D S Y E3 ubiquitin-prot. ligase Praja-1

L P Q E A F E K Y Structural maintenance of chromosomes prot. 3

M P A D T N K A F ER membrane prot. complex subunit 3

L P N D G D E K Y Programmed cell death prot. 2-like

M P I S S H V D L Spermatogenesis-associated prot. 5-like prot. 1

N P N S P S I T Y Enhancer of rudimentary homolog

Q P I N L I F R Y Small nuclear ribonucleoprot. E

F P S I Q A V K I Carboxypeptidase A1

L P A K I L V E F ZW10 interactor

F P E I D L E K Y Dihydrofolate reductase

L P H A P G V Q M Histone deacetylase 2

A V S A V V H E Y Sterol O-acyltransferase 1

M P Y S H P S S Y Nipped-B-like prot.

S A V E E K V S Y KIF1-binding prot.

Y P N G V V V H Y Vam6/Vps39-like prot.

D P F V D R I G Y Nucleoporin NUP188 homolog

V P P S S P Q E L Prot. FAM186A

N V A D L H E K Y Proteasome subunit beta type-5

F P S I Y D V K Y CCR4-NOT transcription complex subunit 8

M P A V K A I I Y Squalene synthase

V V A P I T T G Y Calcyclin-binding prot.

E A F R E H Q Q Y Epithelial-stromal interaction prot. 1

L P L E E A Y R F EH domain-containing prot. 4

E D K Q P C Y I L Twinfilin-1

M A A D D V E E Y Programmed cell death prot. 10

L P S N D S S K F Nucleolar transcription factor 1

L P G E S L T F M Seizure 6-like prot.

T G V R Q V P G F Dermokine

H P L F T Q S Q E S Centrosomal prot. of 97 kDa

L P S L G L S S L D Transmembrane prot. 63B

L P I E N D T Y K Y Syntaxin-binding prot. 3

I P S L N V D C A V Citrate lyase subunit beta-like prot., mitochondrial

L P A D I T E D E F Splicing factor, proline- and glutamine-rich

I P S E V D V E K Y Prot. FAM48A

A S S P S S A H S A Neuron navigator 2

Y P Y N A P T V K F Ubiquitin-conjugating enzyme E2 C

Q A F Q E R L N S Y Nuclear pore complex prot. Nup155

T P I Y E G R T Y Y Plakophilin-4

N L Q T V N V D E N 60S ribosomal prot. L31

M P F P T E E E S V Proto-oncogene vav

L P I E N D V Y K Y Syntaxin-binding prot. 1

I P I A G R D I T Y Actin-related prot. 3B

M P V D P N E P T Y Inhibitor of growth prot. 4

M P F Q R A G V N F SWI/SNF-related reg. of chromatin subfamily A-like prot. 1

M A A T N R P N S I Transitional endoplasmic reticulum ATPase

V A S L S S Q L Q D Myosin-11

A G F A G D D A P R POTE ankyrin domain family member I

L P S P V T A Q K Y Elongation factor 2

F P M T H G N T G F Poly(rC)-binding prot. 2

T A A L I K G E L Y Intraflagellar transport prot.172 homolog

Y A Q D E H L I T F U5 small nuclear ribonucleoprot. 200 kDa helicase

Q D V N G T L V S I Leucyl-cystinyl aminopeptidase

Y P I D P V T Q E F Lymphoid-specific helicase

S P I E F L E N A Y Lanosterol 14-alpha demethylase

E V S S A T N A L R U1 small nuclear ribonucleoprot. A

T P I E E R G D L F Zinc finger prot. 1 homolog

E A F N M I D Q N R Myosin regulatory light polypeptide 9

P G P L G P S A F F Zinc finger prot. 469

G V K A A L L Q L L Cyclin-dependent kinase 13

L P L G N G K A A E E Sodium- and chloride-dependent GABA transporter 3

A L D L Y P E P A F L Putative uncharacterized prot. C17orf82

V L S I S P N C G Y I Macrophage-stimulating prot.receptor

L A L I K I F G A L I Adenosine 3'-phospho 5'-phosphosulfate transporter 2

P T T A E G T S M P I Mucin-17

Q P L D E E L K D A F Sodium/potassium-transporting ATPase subunit alpha-1

A P F Q T S A A M H H Homeobox prot. DLX-5

L P V P N L D P D T Y Sulfite oxidase, mitochondrial

M P V R G P D V E A Y Transmembrane prot. 9B

F S N V M I H V V Q Y Polyprenol reductase

M P K S E V A S S V F Oxysterol-binding prot.-related prot. 11

E A F D E L L A S K Y Regulator of G-prot.signaling 2

N P F D S Q E A K P Y COP9 signalosome complex subunit 2

M A P E R I S G E Q Y Dual specificity mitogen-activated prot.kinase kinase 5

S P L G M P D P H L Y Fc receptor-like A

F P I I I H D E P T Y Pseudopodium-enriched atypical kinase 1

Q P A P S S T S G S Y RNA-binding prot. FUS

Q A A D I D T R S E F Actin-related prot. 2

V P E E G G A T H V Y A-kinase-interacting prot. 1

M P I R E G D T V T L B-cell receptor CD22

F P Q E E A I I D K Y All-trans-retinol 13,14-reductase

L I C N V G A G G P A 60S acidic ribosomal prot. P1

M D S T E P P Y S Q K Elongation factor 1-alpha 1

L P F P D E T H E R Y Glycosylphosphatidylinositol anchor attachment 1 prot.

T P D P K M N A R T Y Splicing factor 3B subunit 1

P M F I V N T N V P R Macrophage migration inhibitory factor

F A N E E G E A Q K F Antigen peptide transporter 1

N P A D S I S H V A Y Transformation/transcription domain-associated prot.

L P I G D V A T Q Y F T-complex prot. 1 subunit eta

L P F D G S P K I T Y HERV-V_19q13.41 provirus ancestral Env polyprot. 1

M P V G P D A I L R Y Large proline-rich prot. BAG6

V V D C T L K L D P I Heterogeneous nuclear ribonucleoprot. D0

T P I Q D N V D Q T Y Germinal center-associated signaling and motility prot.

N F G I G Q D I Q P K 60S ribosomal prot. L7a

L P Y K I T A E E M Y Pre-mRNA branch site prot. p14

E P I Y P E V V H M F Ser/thr phosphatase 2A 56 kDa regulatory subunit gamma

Q P L L I I G K G A A Y 2-hydroxyacyl-CoA lyase 1

M V P P T S G T S T P R Collagen alpha-1(XXVII) chain

M P T G K Q L A D I G Y Cytochrome c oxidase assembly prot. COX14

A S G P P V S E L I T K Histone H1.3

I L G T A G T E E G Q K Quinone oxidoreductase

L P S Q E D M P H N Q F Lysosomal-associated transmembrane prot. 5

N N A S T D Y D L S D K 60S ribosomal prot. L3

F P A V G E P N I Q Q Y UDP-N-acetylgluc.peptide N-acetylglu.transf. 110 kDa subunit

Y P Y D G I H P D D L S F Tyrosine-prot. kinase Lyn

A G K S G S A L E L S V E Prot. phosphatase 1B

Y F Q I N Q D E E E E E D E D 60S ribosomal prot. L22

H K E L A P Y D E N W F Y T R 40S ribosomal prot. S19

**High binding peptides associated with HLA-B*35:62 (origin LCL 721.221cells)**

Peptide position

1 2 3 4 5 6 7 8 9 10 11 12 13 14 15 16 17 18 19 20 21

Ligands Source

E A F Q L F D R Myosin light polypeptide 6

I P D W F L N R 40S ribosomal prot. S18

A D N L C R K L YEATS domain-containing prot. 2

H P T I I S E S F T-complex prot. 1 subunit delta

V L L A E V Q Q H WD repeat-containing prot. 72

I P N E I I H A L Heterogeneous nuclear ribonucleoprot. M

I G P L G L S P K 60S ribosomal prot. L12

A L Q F L E E V K T-complex prot. 1 subunit zeta

I A I Y E L L F K 40S ribosomal prot. S10

M P A E I V E L H Transcriptional repressor prot. YY1

T G F Q A V T G K Eukaryotic translation initiation factor 2 subunit 2

N A F K E I T T M Transcription initiation factor IIB

N S T F S E I F K Transketolase

Y P V D L G D K F DNA-directed RNA polymerases I, II, and III subunit RPABC3

L P F D K E T G F SRA stem-loop-interacting RNA-binding prot., mitochondrial

K N S S D L K Y R Proto-oncogene tyrosine-prot. kinase ROS

V P K P D L D S Y DNA replication complex GINS prot. SLD5

F D Q L L A E E K Myosin-14

A Y D A T H L V K S-formylglutathione hydrolase

H P I R I A D G Y T-complex prot. 1 subunit epsilon

P A A P I T E I V NACHT, LRR and PYD domains-containing prot. 4

I A S N A G S I A 60S ribosomal prot. L23

P G D P A S D E G D RUN domain-containing prot. 1

S I P G G Y N A L R Ubiquilin-4

Q L S K E A L L K L Ras GTPase-activating prot. SynGAP

E V S S A T N A L R U1 small nuclear ribonucleoprot. A

Y I K G G N S E I K HERV-K_6q14.1 provirus ancestral Gag-Pol polyprot.

T P E T L C H V G V Myelin gene regulatory factor

D C I L D E D H S G P Lymphocyte antigen 75

M G G D I A N R V L R Proliferation-associated prot. 2G4

R T I A Q D Y G V L K Peroxiredoxin-1

P M F I V N T N V P R Macrophage migration inhibitory factor

I P V N E K D T L T Y Histone deacetylase complex subunit SAP30

Y Q A V T A T L E E K Putative 60S ribosomal prot. L13a-like MGC87657

E D E D D E E D F E D Neural Wiskott-Aldrich syndrome prot.

K P M V V L G S S A L Q NADH-ubiquinone oxidoreductase 75 kDa subunit, mitochondrial

K L I S D T I S D A L L WD repeat-cont. planar cell pol.effprot. fritz homolog

R K P D T I E V Q Q M K Moesin

T P L H E A A A K G K Y Tankyrase-2

S V Q P T S E E R I P K Septin-9

R I E P A D A H V L Q K N-alpha-acetyltransferase 50

K Q M V I D V L H P G K 40S ribosomal prot. S24

A A A L E A M K D Y T K Stress-induced-phosphoprot. 1

H P I S S E E L L S L K Prot. C-ets-1

R T A A T L A T H E L R Leucine-rich repeat-containing prot. 47

K F Y N Q V S T P L L R Inter-alpha-trypsin inhibitor heavy chain

N N A S T D Y D L S D K 60S ribosomal prot. L3

G Y V V R I S G G N D K 40S ribosomal prot. S6

L P S Q E D M P H N Q F Lysosomal-associated transmembrane prot. 5

Y Y K V D E N G K I S R Ubiquitin-40S ribosomal prot. S27a

G K G F G F I K L E S R Splicing factor, proline- and glutamine-rich

L P A G W I L S H L E T Y Cytochrome c oxidase subunit 8A, mitochondrial

M L L H S E Q H P G Q L K Pyrroline-5-carboxylate reductase 1, mitochondrial

M E K D D S A Q T R Y I K E3 ubiquitin-prot. ligase RING2

S A Y E F S E T E S M L K Leukotriene A-4 hydrolase

G F V K V V K N K A Y F K 60S ribosomal prot. L5

A T D F V A D R A G T F K Isocitrate dehydrogenase [NADP], mitochondrial

V E E I A P D P S E A K R E3 ubiquitin-prot. ligase RAD18

T K T P G P G A Q S A L R 40S ribosomal prot. S14

A S M Q Q Q Q Q L A S A R Chromatin target of PRMT1 prot.1

A Q V A R P G G D T I F G K Histidine triad nucleotide-binding prot. 1

K K Y E E M N A E I S Q F K Guanylate-binding prot. 6

H G V V P L A T Y M R I Y K 60S ribosomal prot. L21

V V K V A N V S L L A L Y K 40S ribosomal prot. S23

S L E S I N S R L Q L V M K 60S ribosomal prot. L30

S P V A K D V D L E F L A K Transitional endoplasmic reticulum ATPase

A A L R P L V K P K I V K K 60S ribosomal prot. L32

Q I S R L E E R E A E L K K C-Jun-amino-terminal kinase-interacting prot. 4

N Q Q I T H A N N T V S N F K Heat shock prot. 105 kDa

G K V R D K L N N L V L F D K 40S ribosomal prot. S25

S K M T T D E L S V S E N I L Prot. eyes shut homolog

S I Y G E K F E D E N F I L K Peptidyl-prolyl cis-trans isomerase A

S E A V A D R E D D P N F F K Glutamate dehydrogenase

D T G K T P V E P E V A I H R 40S ribosomal prot. S20

P Q D S P G Q A L A G Q A T P E CMT1A duplicated region transcript 15 prot.-like prot.

E V Y Q Q Q Q Y G S G G R G N R Heterogeneous nuclear ribonucleoprot. A/B

N K D I R K F L D G I Y V S E K 60S ribosomal prot. L9

T G A A P I I D V V R S G Y Y K 60S ribosomal prot. L27a

G G K P E P P A M P Q P V P T A 40S ribosomal prot. S3

A Q L G G P E A A K S D E T A A Heat shock prot. beta-1

K L T G K D V N F E F P E F Q L 40S ribosomal prot. S7

S Q S A A V T P S S T T S S T R Proteasomal ubiquitin receptor ADRM1

L V L V G D G G T G K T T F V K GTP-binding nuclear prot.Ran

S T A V K A L T G G I A H L F K Dihydrolipoyl dehydrogenase, mitochondrial

K I L D S V G I E A D D D R L N K 60S acidic ribosomal prot.

A A K V L E Q L T G Q T P V F S K 60S ribosomal prot. L11

S A I N E V V T R E Y T I N I H K 60S ribosomal prot. L31

A L L E R T G Y T L D V T T G Q R K Heterogeneous nuclear ribonucleoprot. Q

T A D T I L N T L Q N I S E G L V V Adenylosuccinate lyase

T V A G G A W T Y N T T S A V T V K 60S ribosomal prot. L37a

N L A S R P Y S L H A H G L S Y E K Coagulation factor V

K A V P K E D I Y S G G G G G G S R Heterogeneous nuclear ribonucleoprot. A0

K L N I A R N E Q D A Y A I N S Y T R Acetyl-CoA acetyltransferase, mitochondrial

G R S I S L Y Y T G E K G Q N Q D Y R Nucleolin

N K P G P N I E S G N E D D D A S F K Eukaryotic translation initiation factor 5B

S A A Q A A A Q T N S N A A G K Q L R Plasminogen activator inhibitor 1 RNA-binding prot.

N Q Q E I P S Y L N D E P P E G S M K UPF0444 transmembrane prot. C12orf23

A M E G I F I K P S V E P S A G H D E L Stromal cell-derived factor 2-like prot. 1

E A H Q L F L E P E V L D P E S V E L K Flap endonuclease 1

N G T Q V H G T I T G V D V S M N T H L K Small nuclear ribonucleoprot. Sm
