# Supplementary material for: Understanding the obstacle of incompatibility at residue 156 within HLA-B*35 subtypes
Source: Immunogenetics. 2016 Jan 12;68:247–60. doi: 10.1007/s00251-015-0896-4 (PMC4799800; doi:10.1007/s00251-015-0896-4)
Supplement: Supplementary file 2 — (DOCX 23 kb) [file 251_2015_896_MOESM2_ESM.docx]

**Supplementary 2**

**Table S1** Sequence and origin of shared peptides

| In absence of TPN | | | | |
| --- | --- | --- | --- | --- |
|  |  | LCL 721.220/  HLA-B*35:01 | LCL 721.220/  HLA-B*35:08 | LCL 721.220/  HLA-B*35:62 |
| AL S T G E K G F GY K | Pept.-prolyl cis-trans isomer. A | HB | HB | LB |
|  |  |  |  |  |
| In presence of TPN | | | | |
|  |  | LCL 721.221/  HLA-B*35:01 | LCL 721.221/  HLA-B*35:08 | LCL 721.221  / HLA-B*35:62 |
| L P Q E A F E K Y | Structural maintenance of chromosomes protein 3 | LB/HB | LB | LB |
| T P I Q D N V D Q T Y | Germinal center-associated signaling and motility protein | HB | HB | LB |
| L P N D G D E K Y | Programmed cell death protein 2-like | HB | LB/HB | LB |
| Y P N G V V V H Y | Vam6/Vps39-like protein | HB | HB | LB |
| L P F D K E T G F | SRA stem-loop-interacting RNA-binding protein, mitochondrial | HB | LB/HB | HB |
| F P N A I E H T L | Ubiquitin-like modifier-activating enzyme 1 | HB | LB/HB | LB |
| V P E E G G A T H V Y | A-kinase-interacting protein 1 | HB | LB/HB | LB |
| H A V S E G T K A V T K Y T S A | Histone H2B type 1-J | LB/HB | LB | LB |
| I G P L G L S P K | 60S ribosomal protein L12 | LB | LB | LB/HB |
| F P D E T H E R Y | Glycosylphosphatidylinositol anchor attachment 1 protein | HB | HB | LB |

Sequence and origin of shared peptides. Peptides sequences are depicted in N-terminal to C-terminal orientation. Annotations: LB - low binding peptides; HB - high binding peptid
