# Supplementary material for: Understanding the obstacle of incompatibility at residue 156 within HLA-B*35 subtypes
Source: Immunogenetics. 2016 Jan 12;68:247–60. doi: 10.1007/s00251-015-0896-4 (PMC4799800; doi:10.1007/s00251-015-0896-4)
Supplement: Supplementary file 3 — (DOCX 226 kb) [file 251_2015_896_MOESM3_ESM.docx]

**Supplementary 3**

**Fig. S1**

| **a** | **b** |
| --- | --- |
|  |  |
| **c** | **d** |
|  |  |
| **e** | **f** |
|  |  |
| **g** | **h** |
|  |  |
|  |  |

**Fig. S1** Frequencies of AAs at p2 and pΩ. The x axis represents the AA residues at p2and pΩ. The y axis represents the percentage prevalence of individual AAs. Black, grey or crossed bars represent the alleles HLA-B*35:01, B*35:08 or B*35:62.**a** and **b** Frequencies of AAs occurring at p2 in LB peptides. **c** and **d** Frequencies of AAs occurring at p2 in HB peptides. **e** and **f** Frequencies of AAs occurring at pΩ in LB peptides. **g**and **h** Frequencies of AAs occurring at pΩ in HB peptides. Pro was the most frequently occurring AA at p2 position among the HB and LB peptides. However, HLA-B*35:62 is preferentially anchored by Ala at p2 in the absence of TPN. All HLA-B*35/156 variants are predominantly anchord by Tyr, Phe, Leu or Lys at pΩ.
